# ABRAXAS1 orchestrates BRCA1 activities to counter genome destabilizing repair pathways—lessons from breast cancer patients

**DOI:** 10.1038/s41419-023-05845-6

**Published:** 2023-05-17

**Authors:** Juliane Sachsenweger, Rebecca Jansche, Tatjana Merk, Benedikt Heitmeir, Miriam Deniz, Ulrike Faust, Cristiana Roggia, Andreas Tzschach, Christopher Schroeder, Angelika Riess, Helmut Pospiech, Hellevi Peltoketo, Katri Pylkäs, Robert Winqvist, Lisa Wiesmüller

**Affiliations:** 1 Department of Obstetrics and Gynecology, Ulm University, Ulm, Germany; 2 Laboratory of Cancer Genetics and Tumor Biology, Translational Medicine Research Unit, Biocenter Oulu, University of Oulu, Oulu, Finland; 3 Institute of Medical Genetics and Applied Genomics, University of Tübingen, Tübingen, Germany; 4 Institute of Human Genetics, Medical Center - University of Freiburg, Faculty of Medicine, University of Freiburg, Freiburg, Germany; 5 Leibniz Institute on Aging - Fritz Lipmann Institute, Jena, Germany; 6 Faculty of Biochemistry and Molecular Medicine, University of Oulu, Oulu, Finland; 7 Laboratory of Cancer Genetics and Tumor Biology, Northern Finland Laboratory Centre, Oulu, Finland

**Keywords:** Breast cancer, Mechanisms of disease

## Abstract

It has been well-established that mutations in *BRCA1* and *BRCA2*, compromising functions in DNA double-strand break repair (DSBR), confer hereditary breast and ovarian cancer risk. Importantly, mutations in these genes explain only a minor fraction of the hereditary risk and of the subset of DSBR deficient tumors. Our screening efforts identified two truncating germline mutations in the gene encoding the BRCA1 complex partner ABRAXAS1 in German early-onset breast cancer patients. To unravel the molecular mechanisms triggering carcinogenesis in these carriers of heterozygous mutations, we examined DSBR functions in patient-derived lymphoblastoid cells (LCLs) and in genetically manipulated mammary epithelial cells. By use of these strategies we were able to demonstrate that these truncating *ABRAXAS1* mutations exerted dominant effects on BRCA1 functions. Interestingly, we did not observe haploinsufficiency regarding homologous recombination (HR) proficiency (reporter assay, RAD51-foci, PARP-inhibitor sensitivity) in mutation carriers. However, the balance was shifted to use of mutagenic DSBR-pathways. The dominant effect of truncated ABRAXAS1 devoid of the C-terminal BRCA1 binding site can be explained by retention of the N-terminal interaction sites for other BRCA1-A complex partners like RAP80. In this case BRCA1 was channeled from the BRCA1-A to the BRCA1-C complex, which induced single-strand annealing (SSA). Further truncation, additionally deleting the coiled-coil region of ABRAXAS1, unleashed excessive DNA damage responses (DDRs) de-repressing multiple DSBR-pathways including SSA and non-homologous end-joining (NHEJ). Our data reveal de-repression of low-fidelity repair activities as a common feature of cells from patients with heterozygous mutations in genes encoding BRCA1 and its complex partners.

## Introduction

Deleterious changes in most high and moderate penetrance breast and ovarian cancer (HBOC) risk genes are causally linked with deficiencies in the DSB-response and repair by HR [1, 2]. Changes in the HBOC genes *BRCA1* and *BRCA2* explain a quarter of the familial risk [2]. Screening other HR genes identified additional definitive HBOC genes, including *BARD1, PALB2* and *BRIP1*, all three encoding BRCA1-binding proteins [3]. While PALB2 serves HR by bridging the coiled-coil domain of BRCA1 with BRCA2 [4, 5], BARD1 interacts with BRCA1´s RING domain to aid BRCA1 in counteracting 53BP1 on DNA ends [6]. Through its BRCA1 C-Terminal (BRCT) domains, BRCA1 forms mutually exclusive complexes with the pSPxF-motifs containing proteins ABRAXAS1 (alias ABRA1, CCDC98, FAM175A) [7–9], BRIP1 (BRCA1 interacting protein C-terminal helicase 1, alias BACH1, FANCJ) [10, 11] and CTIP (C-terminal binding protein-interacting protein, alias RBBP8) [12, 13]. These three CDK-mediated, phosphorylation-dependent interactions result in formation of BRCA1-A to -C complexes in S- and G2-phase of the cell cycle [14].

Of particular interest for this work, ABRAXAS1 serves as adapter of BRCA1-A complex components BRCA1, RAP80, MERIT40, BRCC45 and BRCC36 [9, 15–17]. In this way, BRCA1-A (i) accumulates BRCA1 at DSBs via RAP80-mediated recognition of Lys63-linked ubiquitin (K63Ub) adducts in the damaged chromatin [9, 15, 18], (ii) terminates the DDR via BRCC36-mediated K63Ub deubiquitinase (DUB)-activity [19] and (iii) prevents over-resection through sequestration of BRCA1 to the flanks of the DSBs, thereby depleting the local pool of BRCA1 available to enhance DNA end-resection by BRCA1-C [20]. In BRCA1-C, CTIP and MRE11–RAD50–NBS1 (MRN) initiate end-resection at DSBs and therefore HR [21, 22]. There has been a debate on whether BRCA1-A is necessary to ensure proper execution of HR [7, 9, 15, 18, 23] or to prevent excessive HR through antagonizing DSB end-resection [24, 25]. Nevertheless, there is a general consensus on that the localization of the BRCA1-A complex at DNA damage sites is crucial for repair, DDR and genome stability.

*ABRAXAS1* germline variants have been described in Northern Finnish high-risk breast cancer families [23] and in the germline of Non-Finnish cancer patients [1, 26–29]. Yet, a case-control screening study of *ABRAXAS1* missense and non-coding mutations in non-isolated populations of the Breast Cancer Family Registry did not reveal significant association with breast cancer risk [30]. Here, we identified and functionally characterized two *ABRAXAS1* germline mutations in early-onset breast cancer patients from Germany: *ABRAXAS1* c.1106dup, previously identified in the germline of two ovarian cancer patients of a US-Swedish cohort with family history of the disease [1], a US triple-negative breast cancer patient [27] and three further cancer patients [26, 28, 29] but not in the Flossies project of ~10000 cancer-free women >70 (https://whi.color.com/). *ABRAXAS1* c.577C>T was newly identified in patients and recorded once in the Flossies project. Our analysis of functions associated with these *ABRAXAS1* variants revealed a dominant effect of the two truncated ABRAXAS1 proteins on de-repression of the mutagenic DSBR-pathway SSA, yet in different ways, namely by a shift of BRCA1 from BRCA1-A into -C complexes in case of c.1106dup and enhanced formation of the total number of MRE11 complexes as part of excessive stress signaling in case of c.577C>T.

## Results

### Comparative analysis of BRCA1-A complex formation in cells from early-onset breast cancer patients with truncating *ABRAXAS1*-mutations

**Fig. 1:**
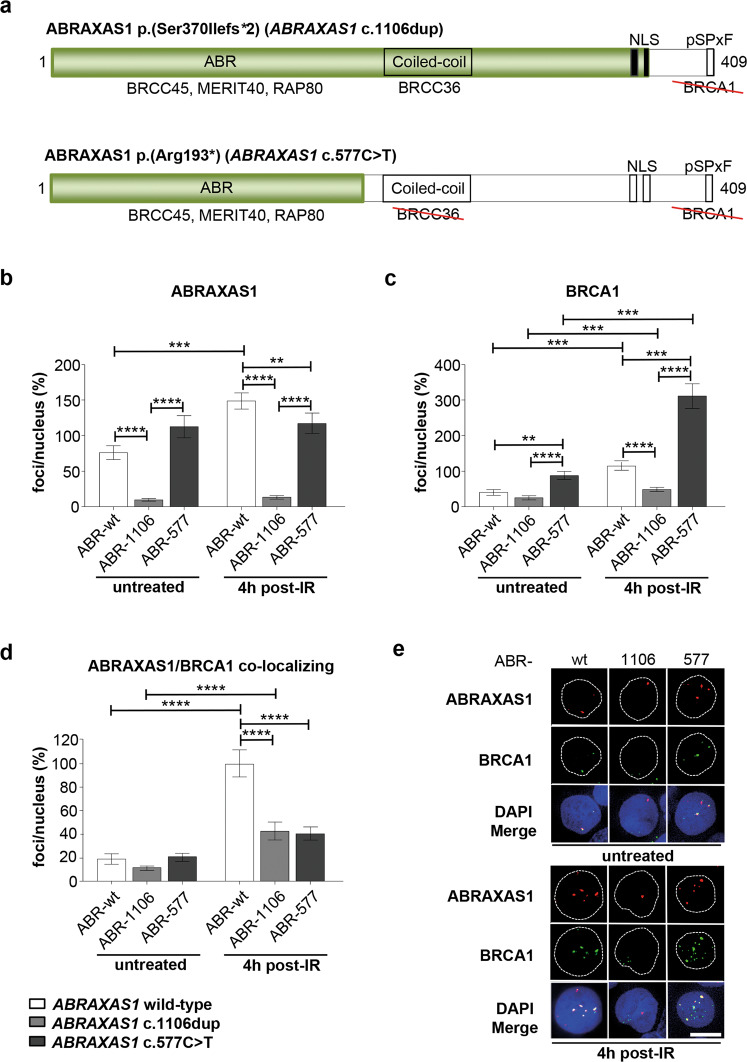
Analysis of ABRAXAS1- and BRCA1-foci accumulation in response to IR. Wild-type *ABRAXAS1* (ABR-wt: white), *ABRAXAS1* c.1106dup-mutated (ABR-1106: light grey) and *ABRAXAS1* c.577C>T-mutated (ABR-577: dark grey) LCLs were either exposed to γ-irradiation with a dose of 2 Gy (IR) followed by cultivation for 4 h or left untreated. Foci per nucleus were scored by automated quantification of 50 nuclei from three independent experiments each and normalized to the mean foci numbers per nucleus calculated from irradiated wild-type LCLs (ABR-wt and external control BR-0968) measured on the same day, which were defined as 100%. GraphPad Prism software was used for graphic presentation and calculation of statistically significant differences via Kruskal–Wallis test followed by two-tailed Mann–Whitney *U* test comparing mean values in ABR-wt, ABR-1106 and ABR-577 under untreated or IR-treated conditions. Columns show mean values; *n* = 150; bars, SEM; **P < 0.01, ***P < 0.001, ****P < 0.0001. **a** Schematic drawings visualizing the truncated mutant proteins ABRAXAS1 p.(Ser370Ilefs*2) encoded by *ABRAXAS1* c.1106dup with deletion of the C-terminal BRCA1-interaction site (comprising a SPTF motif, amino acids 406–409, [23]) and of ABRAXAS1 p.(Arg193*) encoded by *ABRAXAS1* c.577C>T additionally devoid of the BRCC36-interaction site (amino acids 200–270, [65]). Both proteins retain most of the ABR domain mediating interactions with BRCC45, MERIT40 and RAP80. Green, truncated proteins; NLS nuclear localization signal, pSPTF phosphoserine motif that binds to the BRCT domains of BRCA1 [23]. **b** Graphic presentation of ABRAXAS1-foci per nucleus (absolute values corresponding to 100%: 4.2). **c** Graphic presentation of BRCA1-foci per nucleus (absolute values corresponding to 100%: 5.6). **d** Graphic presentation of ABRAXAS1/BRCA1 co-localizing foci (absolute values corresponding to 100%: 4.8). **e** Representative images of ABRAXAS1 (red) and BRCA1 (green) in DAPI-stained nuclei (blue). The scale bars here and further on represent 10 μm.

We studied DDRs in cells from heterozygous *ABRAXAS1*-mutation carriers to understand whether breast cancer-associated mutations in the gene encoding the BRCA1 complex partner ABRAXAS1 entail similar phenotypic changes as described for *BRCA1*-mutations. To this end we engaged LCLs ABR-1106, ABR-577 and ABR-wt from two German mutation carriers (c.1106dup, c.577C>T) and one healthy family member with wild-type *ABRAXAS1*, respectively (Supplementary Table S1). In parallel, we analyzed LCL BR-0968 as representative external healthy wild-type control from a previous study [31]. Strikingly, in both *ABRAXAS1*-mutation carriers breast cancer was diagnosed at early age (25 years). Both mutations caused truncations, thereby deleting the C-terminal BRCA1-binding region in case of *ABRAXAS1* c.1106dup, p.(Ser370Ilefs*2) and the BRCA1- plus BRCC36-interaction sites in case of *ABRAXAS1* c.577C>T, p.(Arg193*) (Fig. 1a). Western blotting engaging mAbs recognizing the C-terminal amino acids 394–408 demonstrated reduction of full-length ABRAXAS1 protein down to on average 60% in cells from heterozygous mutation carriers when compared to the controls (Supplementary Fig. S1a). Concomitantly, BRCA1 levels showed a trend of downregulation to ~70% in *ABRAXAS1*-mutated LCLs (Supplementary Fig. S1b).

To understand whether these changes in protein levels also impact on formation of DNA damage-associated protein complexes, we performed quantitative immunofluorescence microscopy of nuclear proteins in cells 4 h after treatment with 2 Gy ionizing radiation (IR), when ABRAXAS1 signals peaked (data not shown). ABRAXAS1 showed tenfold reduced foci numbers in ABR-1106 compared with ABR-wt independently of IR (Fig. 1b). ABR-577 displayed normal ABRAXAS1-foci numbers pre-IR and 21% reduction post-IR. IR-induced foci increases seen for ABR-wt were neither detectable for ABR-1106 nor ABR-577. Surprisingly, BRCA1-foci were two- and threefold elevated in ABR-577 compared with ABR-wt before and after IR (Fig. 1c). Differently, ABR-1106 formed BRCA1-foci twofold less efficiently post-IR. Yet, IR-induced formation was observed in all three cell types. Thus, despite decreases in overall protein levels, ABRAXAS1-foci numbers were constitutively high in ABR-577 and BRCA1-foci numbers also elevated. In ABR-1106 ABRAXAS1 and to a lesser extent also BRCA1-foci formation were compromised.

**Fig. 2:**
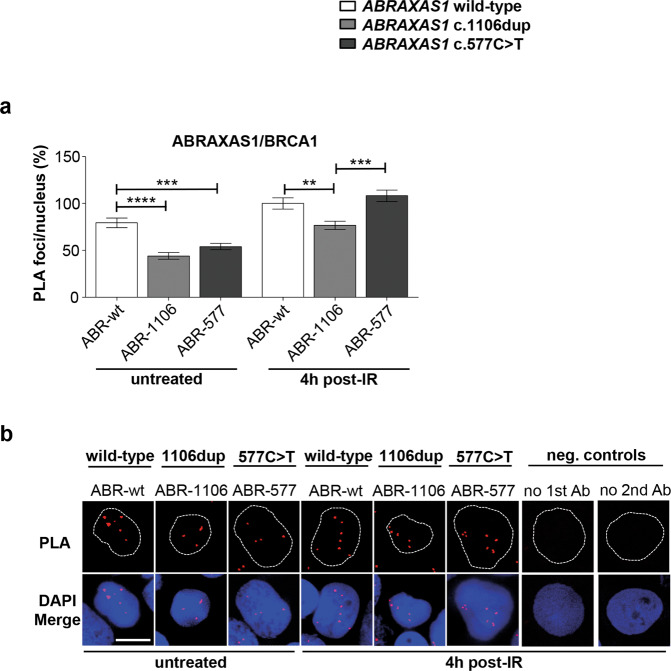
Analysis of BRCA1 and ABRAXAS1 associations. Association between BRCA1 and ABRAXAS1 was examined in LCLs ABR-wt, ABR-1106 and ABR-577 with wild-type *ABRAXAS1* (white columns), with *ABRAXAS1* c.1106dup (light grey column) and *ABRAXAS1* c.577C>T (dark grey column), respectively, via PLA. LCLs were either exposed to IR and re-cultivated for 4 h or left untreated. PLA-foci were scored by automated quantification of 100 nuclei for each time-point in three independent experiments each and normalized to the mean PLA-foci values of the wild-type control ABR-wt 4 h post IR (absolute values corresponding to 100%: PLA, 7.3). Columns show mean values; *n* = 300; bars, SEM; **P < 0.01, ***P < 0.001; ****P < 0.0001. Statistically significant differences were calculated via Kruskal–Wallis test followed by two-tailed Mann–Whitney *U* test comparing mean values for different LCLs. **a** Graphic presentation of ABRAXAS1/BRCA1 PLA-foci per nucleus (%). **b** Representative images of PLA-foci (red) using primary antibodies anti-BRCA1 (mouse) and anti-ABRAXAS1 (rabbit) in DAPI-stained nuclei (blue). Negative controls without the primary (1st) and secondary (2nd) antibody (Ab), respectively, were performed to validate the specificity of the stainings.

When inspecting foci overlays, co-localization of ABRAXAS1- with BRCA1-foci was readily noticeable in ABR-wt, and showed a fivefold increase post-IR (Fig. 1d, e). In both *ABRAXAS1*-mutated LCLs we observed a fraction of BRCA1-foci without ABRAXAS1 co-localization, and colocalizing foci post-IR amounted to only ~40% of the ones in ABR-wt. To directly count multi-protein complexes containing both BRCA1 and ABRAXAS1, named BRCA1-A [32], we engaged PLA. As depicted in Fig. 2, PLA-foci marking association of ABRAXAS1 and BRCA1 were reduced down to half in ABR-1106 and ABR-577 compared to untreated wild-type cells. In IR-treated cells PLA-foci were reduced by 23% in ABR-1106 but not ABR-577. Differences between PLA and co-localization data have been observed before and may be due to the higher sensitivity and spatial resolution of PLA [33]. Altogether, our data suggest compromised BRCA1-A complex formation at DSBs in *ABRAXAS1* c.1106dup-mutated cells, and to some extent also in *ABRAXAS1* c.577C>T-mutated cells despite excessive BRCA1-foci numbers.

### Aberrant DSBR-pathway usage in cells with truncating *ABRAXAS1*-mutations

**Fig. 3:**
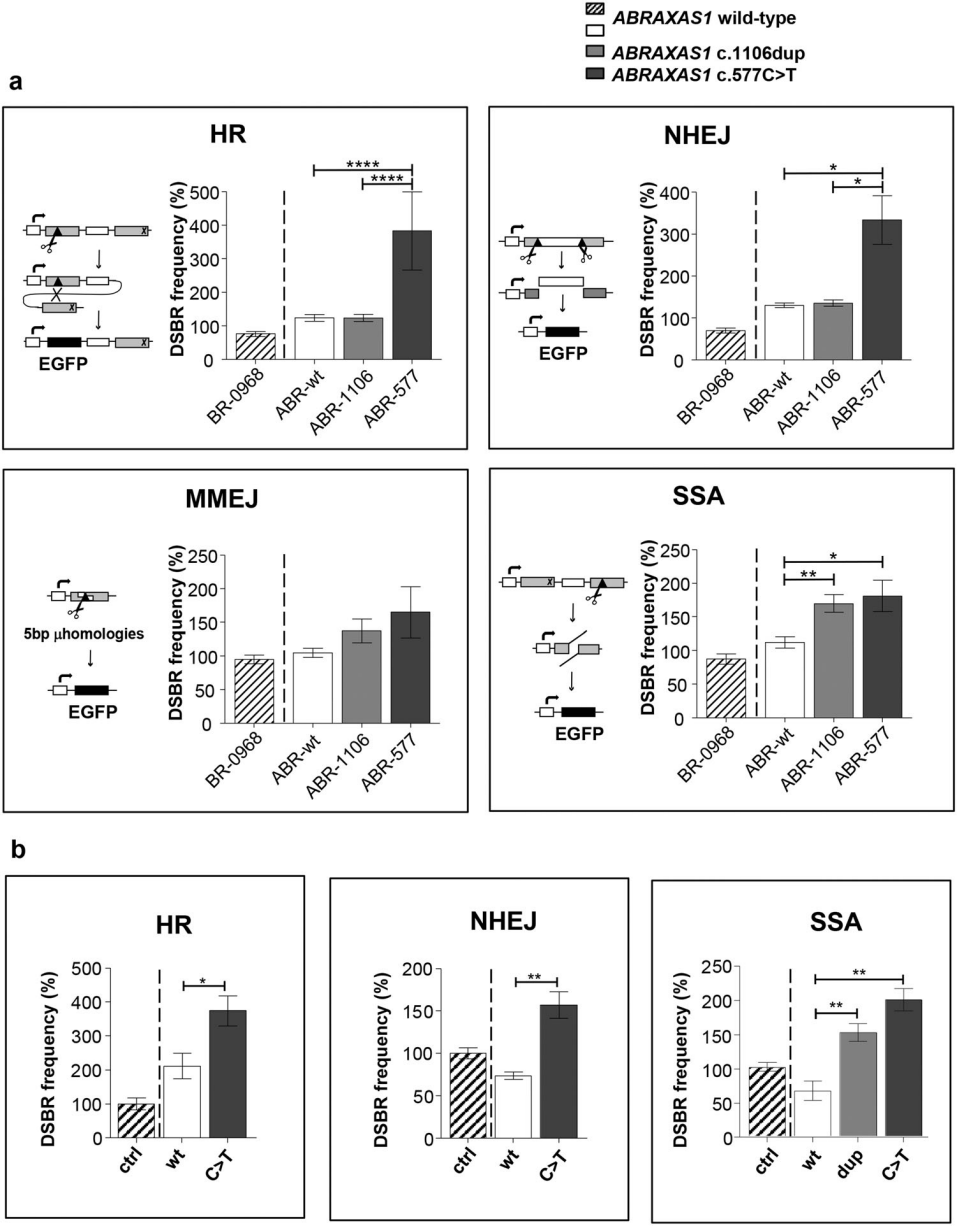
DSBR pathway usage in cells expressing mutated versus wild-type *ABRAXAS1*. Repair events were analyzed by flow cytometry 48 h after transfection of LCLs with EGFP-based and pathway-specific DSBR reporter plasmids plus I-*Sce*I meganuclease expression plasmid. Percentages of EGFP-positive cells in the live cell population were individually corrected for transfection efficiencies obtained in split culture samples transfected with the same plasmid mixture but replacing a filler plasmid by wild-type EGFP expression plasmid (triplicates for repair events and triplicates for transfection efficiencies each). Resulting DSBR frequencies were normalized to the mean frequencies for controls measured on the same day, namely for both wild-type *ABRAXAS1* cell lines in (a) and for wild-type *ABRAXAS1* cells transfected with empty vector in (b). These reference values were defined as 100% (corresponding to 3.7 × 10^−4^ for HR, 3.9 × 10^−3^ for NHEJ, 5.3 × 10^−4^ for MMEJ, 2.5 × 10^−3^ for SSA). Columns show DSBR frequencies; bars, SEM; GraphPad Prism software was used for graphic presentation and calculation of statistically significant differences via Kruskal–Wallis-test followed by two-tailed Mann–Whitney *U* test. *P < 0.05, **P < 0.01, **** *P* < 0.0001; **a** DSBR in heterozygously *ABRAXAS1-*mutated and wild-type individuals. LCLs from two wild-type *ABRAXAS1* individuals (ABR-wt: white columns and external control BR-0968: hatched), one *ABRAXAS1* c.1106dup (ABR-1106: light grey) and one *ABRAXAS1* c.577C>T (ABR-577: dark grey) mutation carrier were transfected with a DNA mixture containing reporter constructs for analysis of HR, NHEJ, MMEJ or SSA (schematically drawn on the left side of each panel [80, 79]). Statistically significant differences were calculated between the mean values in ABR-wt, ABR-1106 and ABR-577. *n* = 15 from five independent experiments; **b** DSBR after ectopic expression of *ABRAXAS1* variants. LCL ABR-wt cells were co-transfected with plasmid mixtures for HR, NHEJ or SSA measurements including I-*Sce*I meganuclease expression plasmid (see a) plus expression plasmids for wild-type *ABRAXAS1* (wt: white), the mutated variants (dup, *ABRAXAS1* c.1106dup: light grey; c.577, *ABRAXAS1* c.577C>T: dark grey) or empty vector (ctrl: hatched). Statistically significant differences were calculated between the mean values in cells expressing exogenous wild-type versus mutated ABRAXAS1. *n* = 6–9 from two to three independent experiments.

Next, we investigated whether imbalanced BRCA1-A complex formation in *ABRAXAS1*-mutated cells impacted on DSBR-pathway usage as previously described for cells from predisposed individuals with heterozygous *BRCA1-* or *BRCA2*-mutations [34–36]. Therefore, we employed EGFP-based reporter substrates enabling specific analyses of DSBR by HR, NHEJ, MMEJ and SSA (Fig. 3a). BRCA1-A complex is known to regulate HR [24]. ABR-577 displayed threefold increased frequencies of HR, however, also of the competing NHEJ-pathway, when compared with the wild-type control. In contrast, HR as well as NHEJ were unaltered in ABR-1106. MMEJ measurements did not reveal a significant change in the *ABRAXAS1*-mutated cells. Intriguingly however, SSA-frequencies were twofold enhanced in both *ABRAXAS1* c.1106dup- and c.577C>T-mutated cells.

To test whether the changes in DSBR-pathway usage can indeed be explained by the truncated ABRAXAS1 proteins, we ectopically expressed them in comparison with wild-type protein in control cells (ABR-wt) (Supplementary Fig. S2a). Expression of both mutant proteins, encoded by *ABRAXAS1* c.1106dup and c.577C>T, stimulated SSA twofold compared with wild-type (Fig. 3b). We also observed a twofold increase in HR- and NHEJ-frequencies when ectopically expressing c.577C>T-encoded ABRAXAS1, altogether, mimicking the differences seen with mutation carrier versus control cells. Moreover, we performed DSBR measurements in the chromatin context after ectopic expression of *ABRAXAS1* variants together with meganuclease I-*Sce*I cleaving a chromosomally integrated reporter. The results show that homologous repair, RAD52-dependent SSA, in particular [37], rose 1.4- to 1.5-fold in cells expressing mutated *ABRAXAS1* (Supplementary Fig. S2b, c). These data excluded the possibility that the differences observed with LCLs were due to clonal particularities demonstrating true effects of the truncated ABRAXAS1 proteins.

To interrogate a key step during HR [38], we scored formation of RAD51 nucleoprotein filaments by immunofluorescence microscopy. While we observed that RAD51-foci numbers rose in all IR-treated cells, we did not observe a statistically significant difference in *ABRAXAS1*-mutated versus wild-type cells (Supplementary Fig. S3a, left panel, and b). However, basal RAD51-foci numbers were reduced in ABR-577 down to 26% compared with ABR-wt. Loss of HR function has been connected with hypersensitivity to poly(ADP-ribose)polymerase (PARP) inhibition [39, 40]. Measurements of the concentration-dependent cell viabilities of LCLs following PARP-inhibitor treatment (1,5-isoquinolinediol, IQD) did not reveal a statistically significant change of IC50-values in *ABRAXAS1*-mutated cells compared with the wild-type (Supplementary Fig. S4a). For comparison, when analyzing *BRCA2*-mutated cells, we observed increased sensitivity in carriers of bi-allelic but again not mono-allelic mutation carriers (Supplementary Fig. S4b). Altogether and as similarly noticed in previous studies with LCLs from heterozygous *BRCA1-*, *BRCA2-* and *PALB2*-mutation carriers [31, 34, 36], mono-allelic mutation of *ABRAXAS1* did not cause a defect in HR but de-repression of SSA.

### Aberrant DDRs in cells with *ABRAXAS1* c.577C>T

**Fig. 4:**
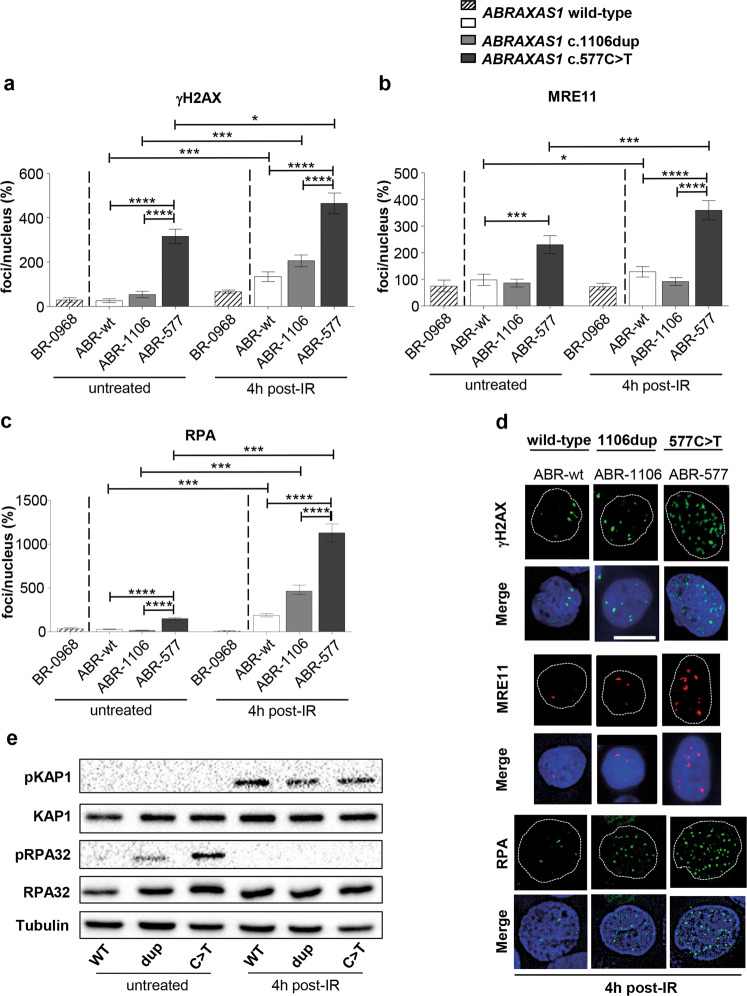
Monitoring replication stress and DSB responses. Wild-type *ABRAXAS1* (ABR-wt: white and external control BR-0968: hatched), *ABRAXAS1* c.1106dup (ABR-1106: light grey) and *ABRAXAS1* c.577C>T (ABR-577: dark grey) LCLs were IR-treated and re-cultivated for 4 h (**a**–**d**). Foci per nucleus were scored by automated quantification and normalized to the mean foci numbers per nucleus calculated from irradiated wild-type LCLs (ABR-wt and external control BR-0968) measured on the same day (100%). Calculation of statistically significant differences between mean values in the LCLs ABR-wt, ABR-1106 and ABR-577 via Kruskal–Wallis test followed by two-tailed Mann–Whitney *U* test. Columns show mean values; bars, SEM; *P < 0.05, ***P < 0.001, ****P < 0.0001. **a** γH2AX-foci per nucleus (*n* = 150–200 from three independent experiments; absolute values corresponding to 100%: 7.2). (**b**) MRE11-foci per nucleus (*n* = 100 from two independent experiments; absolute values corresponding to 100%: 1.9). **c** RPA-foci per nucleus (*n* = 100 from two independent experiments; absolute values corresponding to 100%: 10.3). **d** Representative images of foci (γH2AX: green, MRE11: red, RPA: green) in DAPI-stained nuclei (blue). **e** Analysis of RPA32 and KAP1 phosphorylation. Representative western blot (*n* = 2) showing immunodetection of KAP1 phosphorylated on Ser824, RPA32 on Ser33 and total KAP1 as well as RPA32 in control LCL transfected with expression plasmids for wild-type (wt) or mutant *ABRAXAS1* variants (dup, *ABRAXAS1* c.1106dup; C>T, *ABRAXAS1* c.577C>T) followed by 48 h of unperturbed growth (untreated) or 24 h of growth, IR and recultivation for 4 h (4 h post IR). Detection of Tubulin served as loading control. Uncropped western blots are shown in Extended Figure E2.

To provide insight into the mechanism underlying aberrant DSBR-activities in *ABRAXAS1*-mutated cells, we investigated the hallmarks of DDRs by immunofluorescence microscopy [41, 42]. To investigate recruitment of 53BP1 to DSBs and subsequent clearance we performed time course experiments post-IR (Supplementary Fig. S5a, b). Strikingly, focal nuclear signals were consistently elevated in ABR-577 before and after IR-treatment when compared with ABR-1106 (up to 12-fold) as well as with ABR-wt (up to fivefold). Conversely, ABR-1106 formed 53BP1-foci less efficiently than ABR-wt (down to 28%). When focusing on the small number of gigantic 53BP1 bodies, known to shield under-replicated DNA outside of S-phase [43], we obtained a similar pattern (Supplementary Fig. S5c). Next, we scored the DNA damage marker γH2AX, i.e. focal phosphorylation of Ser139 in H2AX. γH2AX-foci were at least sixfold higher in untreated and at least twofold higher in irradiated ABR-577 compared with ABR-wt and ABR-1106 (Fig. 4a, d).

When DNA ends are resected for HR or SSA, the endo-/exonuclease MRE11 is critically involved in initial processing. The resulting single-stranded DNA (ssDNA) is immediately coated by RPA to protect it from degradation [44]. Microscopic analysis indicated threefold elevated MRE11- and ≥twofold elevated RPA-foci levels in ABR-577 post-IR as compared with the other cell types (Fig. 4b–d). Basal MRE11-foci were twofold higher in ABR-577 compared with ABR-wt, RPA-foci ≥fivefold compared with the other cell types. In ABR-1106 MRE11-foci remained at wild-type level regardless of treatment. RPA-foci rose ≥2.5-fold versus wild-type controls post-IR, reaching statistical significance when compared with BR-0968 (****P < 0.0001).

During replication stress the checkpoint kinase ATR phosphorylates Ser33 in RPA while bound to ssDNA [45]. When we monitored phosphorylation of RPA32 at Ser33 by western blotting, we noticed a rise of pRPA32/RPA normalized band intensities in control LCL cells ectopically expressing *ABRAXAS1* c.1106dup or c.577C>T compared to wild-type by on average twofold (Fig. 4e). For comparison, we also analyzed phosphorylation of the ATM substrate KAP1 on Ser824 in these samples [46], showing comparable IR-induced KAP1 phosphorylation upon expression of all three *ABRAXAS1* variants (<10% differences of pKAP1/KAP1). We conclude that ATR rather than ATM signaling was active in cells expressing mutated ABRAXAS1 variants due to endogenous stress.

HR factors can protect replication forks from MRE11-dependent degradation independently of their recombination-activities [6, 47]. Therefore, we analyzed DNA replication stress as a mechanism potentially underlying constitutively enhanced MRE11-activities in ABR-577. To this end we performed DNA fiber spreading assays with sequential incorporation of CldU and IdU interrupted by hydroxyurea (HU) treatment provoking MRE11-dependent attack of nascent DNA (Supplementary Fig. S6a). Measurement of CldU-positive replication track lengths did reveal the expected track shortening upon HU- versus mock-treatment (Supplementary Fig. S6b). Yet, despite elevated MRE11- and RPA-foci in ABR-577, DNA replication tracks were not shortened but elongated (1.3-fold) in cells ectopically expressing *ABRAXAS1* c.577C>T compared to wild-type. Analysis of the percentage of aberrant CldU/IdU replication track ratios did not carve out any differences (Supplementary Fig. S6c). Altogether, these data sets argue against major differences in replication fork stalling or fork degradation.

**Fig. 5:**
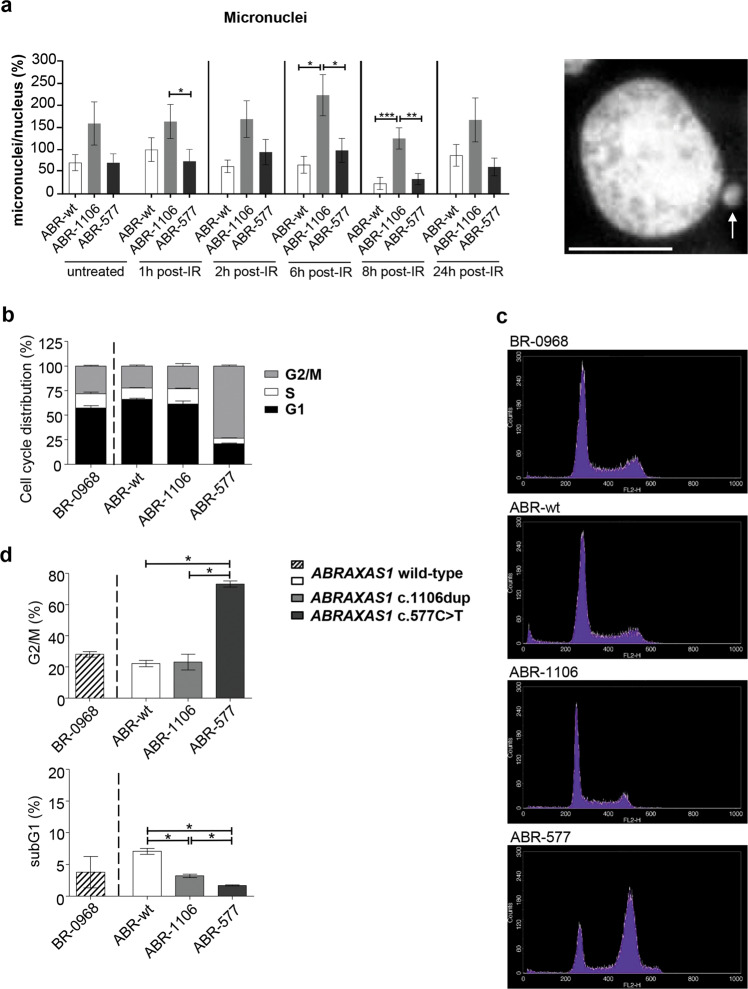
Micronuclei and cell cycle distribution. **a** Quantification of micronucleus formation. LCLs derived from wild-type *ABRAXAS1* individual (ABR-wt) and from the *ABRAXAS1* c.1106dup (ABR-1106) and the *ABRAXAS1* c.577C>T (ABR-577) mutation carriers were exposed to IR and re-cultivated before cells were fixed at the indicated time points. Micronuclei per nucleus were scored and normalized to the mean value of ABR-wt 1 h post IR measured on the same day each. This reference value (100%) represents four micronuclei/100 nuclei. Statistically significant differences between values for LCLs ABR-wt, ABR-1106 and ABR-577 for each treatment condition and pairwise for untreated cells and 1 h post IR were calculated via Kruskal–Wallis test followed by two-tailed Mann–Whitney *U* test. Data points indicate mean values and SEM of 150 nuclei obtained from three experiments. *P < 0.05, **P < 0.01, ***P < 0.001. The image on the right shows a representative microscopic image of a DAPI-stained nucleus with micronucleus at the tip of the white arrow. **b** DNA content was determined flow cytometrically after propidium iodide staining of LCLs from external wild-type control BR-0968, ABR-wt, ABR-1106 and ABR-577. Cell cycle distribution of G1-, S- and G2/M-phases in living cells is graphically presented. **c** Representative histograms of propidium iodide stained LCLs. **d** Statistically significant differences between the mean percentages of cells with G2/M-phase and subG1-DNA content were calculated for ABR-wt, ABR-1106 and ABR-577 via Kruskal–Wallis-test followed by two-tailed Mann–Whitney *U* test (*n* = 4 from two independent experiments); bars, SEM; *P < 0.05.

Given that ABR-577 showed dramatic DDRs already in the untreated state, we tested whether the mutations also affect the genomic stability by quantification of micronuclei post-IR. Strikingly, we noticed up to fivefold higher micronucleus numbers in ABR-1106 with compromised 53BP1-foci formation (Fig. 5a). Next, we analyzed the activation of cell cycle checkpoints [32]. Flow cytometric analysis revealed a similar distribution of ABR-wt and ABR-1106 in the cell cycle phases G1, S and G2/M (Fig. 5b, c). However, ABR-577 showed a three times higher percentage of G2/M-phase cells (Fig. 5c, d). Concomitantly, cell death was reduced in these cells compared to ABR-wt and ABR-1106. However, when we transiently expressed *ABRAXAS1* variants in control LCLs, we observed 1.2- to 1.3-fold G2/M-accumulation in cells expressing c.1106dup versus wild-type for 24 h and 48 h (Supplementary Fig. S7a, b). IR-treatment 24 h post transfection elevated G2/M-DNA contents 1.4-fold in cells expressing both c.1106dup and c.577C>T but not wild-type. Forty-eight hours and 72 h post transfection all cell types showed a similar IR-induced G2/M-accumulation. No significant changes were seen in the percentages of cells with subG1-DNA content (Supplementary Fig. S7c). To specifically detect cell death by apoptosis, we monitored accumulation of the cleaved form of PARP1 by western blotting. We observed appearance in irradiated cells, whereby cleaved PARP signals rose in cells ectopically expressing mutated versus wild-type *ABRAXAS1*, particularly c.577C>T (Supplementary Fig. S7d). Altogether, in LCLs constitutively expressing mutated *ABRAXAS1* we observed pronounced G2/M-accumulation for c.577C>T, in transiently expressing cells minor accumulation for mutated variants, but particularly pronounced IR-induced apoptosis for c.577C>T. In conclusion, ABR-1106 cells showed reduced 53BP1-foci numbers accompanied by micronucleus formation indicating genomic instability, while ABR-577 cells were particular in that they displayed exacerbated DDRs.

Given that RAD51 expression is cell cycle-dependent [48], we scored RAD51-foci in LCL cells ectopically expressing the different ABRAXAS1 proteins for 24 h, i.e. causing minor cell cycle changes. Under these conditions the pattern of RAD51-foci accumulation was similar as seen in patient-derived cells except for threefold lower foci numbers 4 h post IR in cells expressing c.577C>T versus wild-type *ABRAXAS1* (Supplementary Fig. S3a, right panel). These data suggest that RAD51 filament formation at DSBs is compromised in LCLs transiently expressing *ABRAXAS1* c.577C>T, which is rescued in constitutively expressing patient-derived LCLs due to an exacerbated DDR.

### *ABRAXAS1* c.1106dup shifts BRCA1 into the BRCA1-C complex

**Fig. 6:**
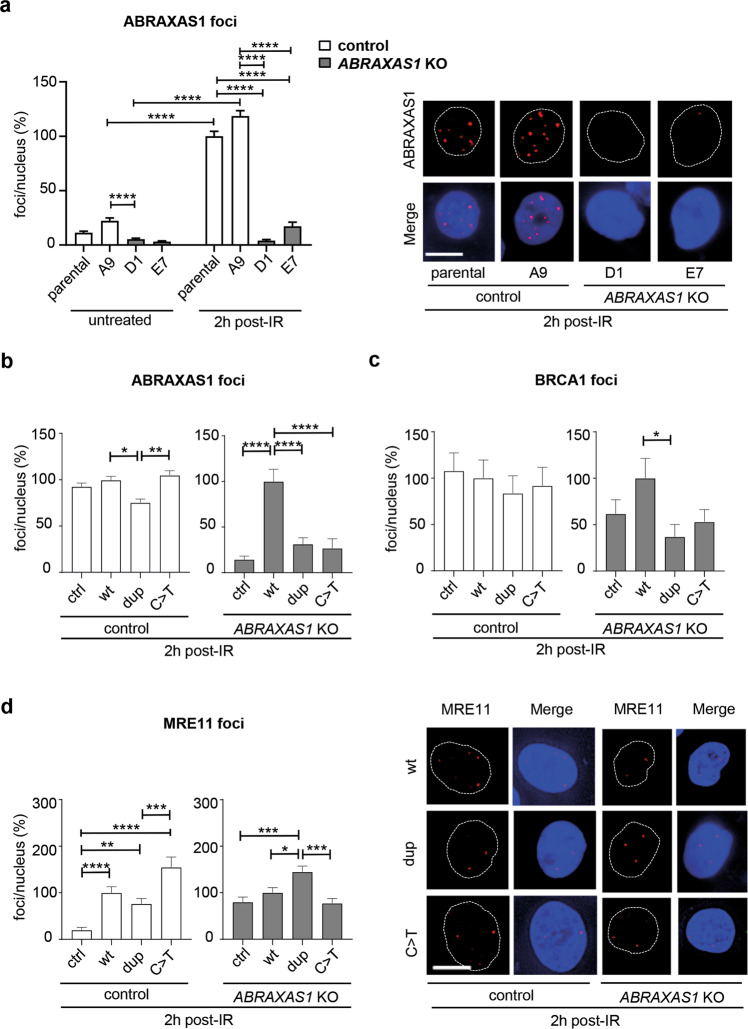
DDR in mammary epithelial cells as a function of ABRAXAS1. Mammary epithelial control MCF10A cells (parental and clone A9) and *ABRAXAS1* KO cells (clones D1 and E7) were nucleofected with expression plasmids for wild-type *ABRAXAS1* (wt), the mutated variants (dup, *ABRAXAS1* c.1106dup; C>T, *ABRAXAS1* c.577C>T) or empty vector (ctrl), irradiated 24 h later with a dose of 2 Gy and re-cultivated for another 2 h. Nuclear foci were scored and normalized to mean foci numbers of controls on the same experimental day (100%). Columns show mean values (*n* = 100–400 from two to four independent experiments); bars, SEM; Kruskal–Wallis test followed by two-tailed Mann–Whitney *U* test; *P < 0.05, **P < 0.01, ***P < 0.001, ****P < 0.0001. **a** ABRAXAS1-foci per nucleus in the different cell types with and without IR (absolute values corresponding to 100%: 8.9 in irradiated parental MCF10A cells). **b**–**d** Foci per nucleus in control (A9) and *ABRAXAS1* KO cells (D1) after expression of different ABRAXAS1 proteins and IR-treatment for 2 h. **b** ABRAXAS1-foci per nucleus (A9 10.3, D1: 3.1 corresponding to 100% in cells expressing wt). **c** BRCA1-foci per nucleus (A9: 1.2, D1: 0.9 corresponding to 100% in cells expressing wt). **d** MRE11-foci per nucleus (A9: 1.9, D1: 2.1 corresponding to 100% in cells expressing wt).

To analyze patient-derived *ABRAXAS1*-mutations in breast epithelial cells, we performed CRISPR/Cas9-based knockout (KO) in immortalized, non-cancer MCF10A cells. *ABRAXAS1-*KO resulted in bi-allelic deletion of 47 bp encompassing the *ABRAXAS1* start codon in clone D1; in clone E7, 11 bp were deleted around the start codon on one allele and 16 bp in combination with a 1410 bp insertion on the second allele (Supplementary Fig. S8a, b). Expression analysis verified complete loss of *ABRAXAS1* mRNA in clone D1; in E7 residual levels were detectable (Supplementary Fig. S8c). When examining ABRAXAS1-foci, we noticed 5- to 11-fold induction post-IR in parental MCF10A and the A9 control clone (Fig. 6a). Consistent with mRNA expression, nuclear ABRAXAS1 signals were at the detection limit in KO clone D1 regardless of IR-treatment, whereas residual ABRAXAS1-foci were detectable in E7 post-IR. Consequently, we chose D1 for analysis of exogenous ABRAXAS1 proteins in the following experiments.

To compare *ABRAXAS1* c.1106dup, c.577C>T and wild-type expression in ABRAXAS1-negative and -positive backgrounds, we ectopically expressed these variants in *ABRAXAS1*-KO- and control MCF10A cells (Supplementary Fig. S8d). Re-expression of wild-type but not mutant ABRAXAS1 reconstituted IR-induced ABRAXAS1-foci formation in KO cells (Fig. 6b, Supplementary Fig. S8e). In ABRAXAS1-positive control cells, we counted fewer ABRAXAS1-foci after expression of the c.1106dup variant versus wild-type or c.577C>T, compatible with an inhibitory effect on foci formation by endogenous ABRAXAS1, already seen in heterozygously mutated LCLs (Fig. 1b). BRCA1-foci numbers post-IR were lower in KO cells ectopically expressing c.1106dup versus wild-type, whereby generally low BRCA1-foci numbers in MCF10A cells did not permit to carve out further statistically significant differences (Fig. 6c). Analysis of MRE11-foci in KO cells post-IR showed that *ABRAXAS1* c.1106dup expression increased foci counts compared with all other samples (Fig. 6d). In ABRAXAS1-positive control cells, maximum MRE11 signals were observed after c.577C>T expression, similar to LCL ABR-577 where this variant stimulated DDRs. In MCF10A cells checkpoint activation after mutant ABRAXAS1 expression was exerted in G1, which reached statistical significance when comparing fold changes between the group of KO cells expressing mutated *ABRAXAS1* (c.1106dup or c.577C>T) with the other KO cells (expressing wild-type or no *ABRAXAS1*, Supplementary Fig. S9a–c).

**Fig. 7:**
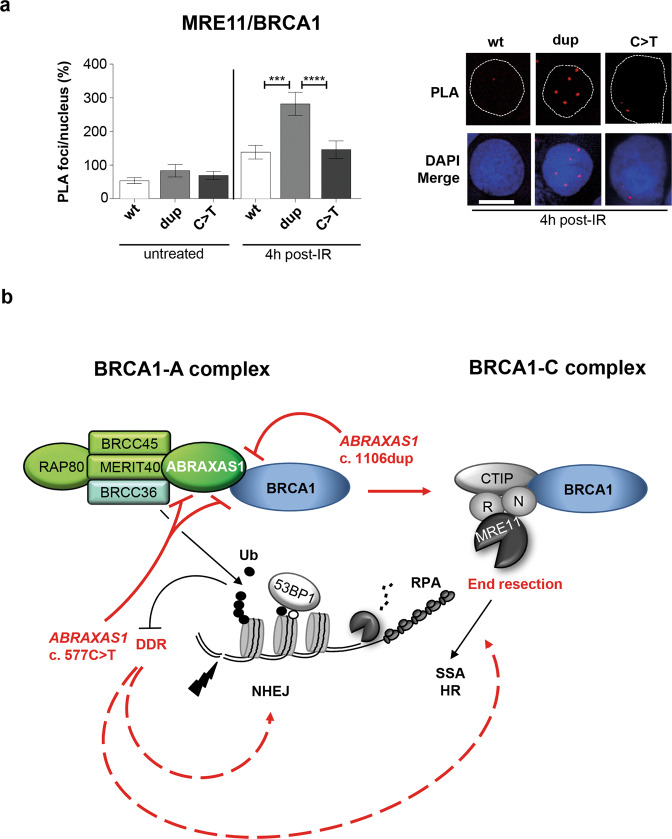
Analysis of BRCA1-C complex formation and model for functional defects of ABRAXAS1 mutant proteins. **a** Analysis of MRE11 and BRCA1 associations. LCL ABR-wt was transfected 24 h before IR with expression plasmids for wild-type (wt) or mutant *ABRAXAS1* variants (dup, *ABRAXAS1* c.1106dup; C>T, *ABRAXAS1* c.577C>T). PLA was performed 4 h post IR. MRE11/BRCA1 PLA-foci were scored by automated quantification of 150 nuclei from three independent experiments and normalized to the mean for the external control transfected with empty vector, which was defined as 100% (0.2). Columns show mean values; *n* = 450; bars, SEM; Kruskal–Wallis test followed by two-tailed Mann–Whitney *U* test; **P < 0.01, ***P < 0.001, ****P < 0.0001. The right panel shows representative images of PLA-foci (red) 4 h post IR using primary antibodies anti-MRE11 (rabbit) and anti-BRCA1 (mouse) in DAPI-stained nuclei (blue). **b** Model for the findings revealing how the truncated ABRAXAS1 mutant proteins differentially deregulate DSBR. See main text for details. In brevity, ABRAXAS1 p.(Ser370Ilefs*2), encoded by *ABRAXAS1* c.1106dup, cannot bind the C-terminal BRCT domains of BRCA1, therefore giving access to CTIP and MRE11-RAD50-NBS1. This causes a shift of BRCA1-A to BRCA1-C complex formation, which spurs end-processing and SSA at DSBs. ABRAXAS1 p.(Arg193*), encoded by *ABRAXAS1* c.577C>T, additionally cannot bind BRCC36, which therefore cannot limit the DSB-induced ubiquitination cascade, entailing excess DNA damage signaling and unspecific DSBR-activities. Red color marks the impact of ABRAXAS1 mutant proteins on biochemical processes underlying the observed changes in DSBR.

Inspired by the fact that MRE11-foci formation was enhanced in IR-treated cells expressing endogenous wild-type *ABRAXAS1* plus c.577C>T in different cell types (Figs. 4b and 6d), we asked whether unleashed MRE11-dependent end-processing (Fig. 4c) and SSA (Fig. 3) were accompanied by BRCA1-association with MRE11 within BRCA1-C [32]. Surprisingly, ectopic expression of *ABRAXAS1* c.577C>T in ABR-wt did not alter the association of MRE11 and BRCA1 versus wild-type. However, expression of c.1106dup doubled MRE11/BRCA1 PLA-foci post-IR (Fig. 7a). Together with our finding that BRCA1-A complex formation was compromised in ABR-1106 (Figs. 1d and 2a), these data suggest a shift of BRCA1 from BRCA1-A to –C in cells expressing ABRAXAS1 devoid of the extreme C-terminus.

## Discussion

In this work, we investigated functional consequences of the *ABRAXAS1*-variants c.1106dup and c.577C>T identified in two German patients with early-onset breast cancer. ABRAXAS1 physically links BRCA1 to the other BRCA1-A complex components, i.e. to BRCC45, MERIT40, BRCC36 and RAP80, thereby accumulating BRCA1 adjacent to DSBs [49]. While BRCA1 as BRCA1-C component is important for MRN/CTIP-dependent initiation of end-processing and therefore homology-directed DSBR [32], its incorporation into BRCA1-A complexes counterbalances these processes [24]. Our work on the two differently truncated proteins dissects defects of the two *ABRAXAS1* variants in mitigating BRCA1-C complex formation via sequestration of BRCA1 in BRCA1-A and in preventing massive DDR and DSBR-activity via BRCC36 incorporation into BRCA1-A (Supplementary Table S2 and Fig. 7b).

ABRAXAS1 is known to bind to the BRCT repeats in BRCA1 via its extreme C-terminal SPTF motif when phosphorylated post-IR by ATM [7–9, 50]. BRCA1 exerts key functions in HR that are lost in pathogenic *BRCA1* variants mutated in the BRCT domain [51]. In our preceding studies engaging cells from heterozygous carriers of pathogenic *BRCA1-*, *BRCA2-* or *PALB2*-mutations, HR was found to be unperturbed [31, 34], while complete loss-of-function was noticeable in bi-allelic carriers of *BRCA1-* or *BRCA2*-mutations [34, 35]. Intriguingly, both mono- and bi-allelic mutation carriers upregulated error-prone SSA and/or MMEJ [34, 35], which was also true for cells expressing BRCA1-binding defective BRIP1 [52]. In the work presented here, we examined whether truncating *ABRAXAS1*-mutations also dysregulate DSBR-pathway usage in heterozygously mutated LCLs. Consistent with the results obtained for *BRCA1*-mutated LCLs, expression of ABRAXAS1 p.(Ser370Ilefs*2), lacking 40 C-terminal amino acids comprising the BRCA1-interaction site, did neither influence HR in the reporter assay nor RAD51 focus formation nor PARP-inhibitor sensitivity. However, one *ABRAXAS1* c.1106dup allele was sufficient to upregulate SSA, indicating haplo-insufficiency for repression of SSA but not for execution of HR.

ABRAXAS1 p.(Ser370Ilefs*2) expression also caused other marked phenotypic changes post-IR, namely reduced BRCA1- accompanied by elevated MRE11-foci numbers in *ABRAXAS1* KO cells as well as compromised BRCA1-A accompanied by enhanced BRCA1-C complex formation in LCLs. Thus, loss of the C-terminal BRCA1-interaction site of ABRAXAS1 is sufficient to de-repress BRCA1-binding to CTIP within the BRCA1-C complex, which is known to accelerate MRN/CTIP-mediated end-resection [53]. In support of this concept we noticed augmented RPA- and reduced 53BP1-foci post-IR. 53BP1 protects DNA ends from resection, antagonizing BRCA1-C-mediated action on DSBs [41]. Our findings agree with a recent report demonstrating that ABRAXAS1 suppresses end-resection and break-induced replication (BIR), a mutagenic subtype of RAD52-mediated HR [54]. Here, we measured enhancement of SSA, which requires long-range end-resection of DSBs and annealing of the resulting ssDNA by RAD52 [55]. Mean MMEJ values increased in cells from mutation carriers, yet arriving at close to statistical significance only when comparing ABR-1106 with the wild-type control BR-0968 (*P* = 0.0512), which may reflect the very short resection length required for MMEJ compared to SSA. HR did not show any increase in ABR-1106 cells, which connects with the decrease of nuclear 53BP1 known to channel post-resection mechanisms towards HR by inhibiting extensive resection required for SSA [56]. At first sight contradictory to these findings, LCLs from carriers with the Finnish high-risk breast cancer susceptibility variant *ABRAXAS1* c.1082 G > A performed SSA and MMEJ at reduced frequencies [57]. However, encoded ABRAXAS1 p.(Arg361Gln) still binds BRCA1 but lacks a functional NLS causing cytoplasmic BRCA1 sequestration, i.e. unavailability for BRCA1-A and BRCA1-C complexes in the nucleus. Our findings indicate that ABRAXAS1 p.(Ser370Ilefs*2) exerts a very different effect on BRCA1. Thus, it can be recruited to DSB sites but no longer sequester BRCA1 into these. This imbalance unleashes end-resection and ultimately low-fidelity repair by SSA [55, 58].

Differently, cells from the *ABRAXAS1* c.577C>T carrier did not show a pathway shift but broad stimulation of multiple DSBR-pathways. The SSA increase might be explained by loss of the BRCA1-binding site in ABRAXAS1 p.(Arg193*) in analogy to the p.(Ser370Ilefs*2)-associated phenotype. The rise in HR is more difficult to explain. Previous studies reported contradictory results on the role of ABRAXAS1 in HR when using a similar reporter substrate as engaged in our work. While Livingston and co-workers found HR stimulation after ABRAXAS1, RAP80 or BRCC36 silencing [24], others observed a moderate decrease after ABRAXAS1 or RAP80 depletion [9, 18]. In this context it is of interest that even HR must be tightly controlled to prevent excess, untimely and/or RAD51-independent repair [38, 55, 59]. Suggesting use of such alternative mechanisms, RAD51-foci numbers did not rise above wild-type level in c.577C>T-positive LCLs despite constitutively increased nuclear foci of multiple DDR proteins, i.e. γH2AX, MRE11, RPA, BRCA1 and 53BP1. Association of BRCA1 with MRE11, i.e. BRCA1-C complex formation, was normal despite elevated MRE11-foci numbers. BRCA1-independent MRE11 complexes were reported to cause excessive end-resection [60] and CTIP binding-defective BRCA1 induces hyperrecombination [61]. Altogether these observations suggest that uncontrolled accumulation of ssDNA may stimulate SSA in *ABRAXAS1* c.577C>T cells.

Distinct from *BRCA1*-mutated cells [34, 35], we did not find elevated MMEJ-frequencies in cells expressing ABRAXAS1 p.(Arg193*). However, reminiscent of the DSBR-pathway shift seen in Fanconi anemia (FA) cells [62] canonical NHEJ was upregulated instead. Noteworthy, we previously found that elevated 53BP1 levels promote NHEJ but not MMEJ in cells from *PALB2*-mutation carriers [31]. Therefore, enhanced 53BP1-foci formation may contribute to elevated NHEJ in cells expressing p.(Arg193*). This truncated version of ABRAXAS1 can neither bind BRCA1 nor the DUB BRCC36, which has been implicated in clearance of the K63Ub-binding protein 53BP1 from DSBs [63, 64].

*ABRAXAS1* c.577C>T cells showed markedly enhanced DDRs already under unperturbed growth conditions. To understand the source of DDRs we performed DNA fiber spreading assays, which excluded enhanced replication fork stalling and degradation of nascent DNA. Different from ABRAXAS1 p.(Ser370Ilefs*2), p.(Arg193*) not only lacks the BRCA1 but also the BRCC36-binding site [36, 65]. The DUB-activity of BRCC36 is necessary to terminate DDRs [32]. Wu and Wang [54] observed that in camptothecin-treated *ABRAXAS1* KO cells (U2OS) BRCC36 is needed to prevent aberrant cleavage of stalled forks and subsequent MRE11-dependent end-processing. Here, we observed that MRE11-foci were upregulated in ABRAXAS1 p.(Arg193*)- versus p.(Ser370Ilefs*2)-expressing cells regardless of treatment, however, only in a wild-type background. These data suggest that p.(Arg193*) synergizes with wild-type ABRAXAS1 in the uncontrolled MRE11 response. Though further work is needed to clarify the precise details, we propose that co-existence of wild-type ABRAXAS1, limiting fast and mutagenic DSBR, and of p.(Arg193*), preventing proper termination of the DDR by BRCC36, generate a vicious cycle of DDR amplification. In line with unleashed DDRs, we noticed a dramatic G2/M-phase arrest in ABR-577 cells, which appears to require constitutive *ABRAXAS1* c.577C>T expression as suggested by minor G2/M-accumulation in transient expression experiments. G2/M-phase accumulation has previously been described in lymphocytes and LCLs from FA patients [66] as well as in RAP80-depleted esophageal cancer cells [67]. Such G2/M phase accumulation provides even another explanation for the SSA rise, as resection is stimulated in this phase of the cell cycle [68]. Others observed G1-arrest in IR-treated fibroblasts [69] and exacerbated DDRs with p53-dependent G1-arrest in hematopoietic stem and progenitor cells from FA patients [70]. In epithelial MCF10A cells, used here for KO-experiments, highly efficient G1-checkpoint control was noticed previously [71]. Here, ABRAXAS1 p.(Arg193*)- and to a lesser extent p.(Ser370Ilefs*2)-expressing cells showed a higher G1-phase percentage as compared with wild-type ABRAXAS1-expressing MCF10A cells. Of relevance for diagnostic approaches [66], the outcome of unrestricted DDRs in *ABRAXAS1*-mutated cells may therefore depend on the cell type.

Even though establishment of specific truncating *ABRAXAS1*-mutations as HBOC pathogenic variants will have to await further screening results, our data indicate deleterious effects on protein functions that have been associated with *BRCA1*-mutated patient cells [34–36]. The key phenotypic change observed in *ABRAXAS1* c.1106dup-expressing cells was a shift of BRCA1 from BRCA1-A to BRCA1-C complexes, which promoted deleterious SSA events and genomic instabilities as deduced from elevated micronucleus formation post-IR. *ABRAXAS1* c.577C>T cells were characterized by excess DDRs and DSBR-activities with low fidelity (Fig. 7b). Recently, functional assays have entered clinical routine for assessment of pathogenicity [72]. In the light of our previous findings, elevated SSA-frequencies can be carved out as common denominator of patient cells with pathogenic defects in various BRCA1 complex partners like PALB2 or BRCA2 and as seen here for truncated ABRAXAS1 proteins. Accordingly, inaccurate DSBR-activities such as SSA may not only cause chromosomal instabilities on the route to cancer formation and provide resistance mechanisms during cytostatic treatment but may also serve as a marker for pathogenicity assessment of breast cancer risk gene variants.

## Materials and methods

### Blood samples and identification of *ABRAXAS1*/*FAM175A* variants

The index patient ABR-1106 and her mother ABR-wt (Supplementary Table S1) were counseled at the University Hospital of Tübingen, Germany and the index patient ABR-577 was counseled at the University Hospital of Freiburg, Germany, according to the guidelines of the German Consortium for HBOC. Written informed consent was obtained for scientific use (BRCA20006 173/2011BO2; HerediCaRe 505/2020BO; HerediVar 838/2021BO2). EDTA blood samples were drawn by venipuncture. Genomic DNA was isolated using the Maxwell®16 Blood DNA Kit (Promega, Flitchburg, USA) and Next-generation sequencing technology applied using an Illumina MiSeq sequencing device (Illumina, San Diego, USA) and the customized diagnostic HaloPlex multigene panel for target enrichment (Agilent, Santa Clara, USA) covering 68 cancer predisposition genes. Subsequent data analysis was done with a diagnostic data analysis pipeline (https://github.com/imgag/ngs-bits and https://github.com/imgag/megSAP). Briefly, raw reads were demultiplexed and saved as FASTQ files. Quality control was done with in-house quality control tools. BWA-MEM2 [73] was used for mapping against GRCh37/38 and alignments were post-processed by various tools e.g. for indel realignment and identification of duplicates [74]. Germline variants were detected using freebayes (https://github.com/freebayes/freebayes). Variants were annotated with Ensembl’s VEP [75] and custom in-house data from previously sequenced patients. Variants were classified according to the ACMG guidelines [76].

#### Lymphoblastoid cell lines (LCLs)

LCLs were generated as previously described [57] and used at passage numbers 6–25 for functional analyses. LCL BR-0968 from a previous study was included as representative external healthy wild-type control [31]. Previously described LCLs [34], namely HA238 with heterozygously mutated *BRCA2* (provided by Medical University Hannover, Germany), GM13023A with biallelic *BRCA2/FANCD1*-mutation (purchased at Coriell Institute, Camden, NJ, USA) and TK6 (provided by Eppendorf University Clinic, Hamburg, Germany) were further engaged for comparative analysis of cell viabilities. LCLs were cultured in RPMI 1640 medium (Gibco/Invitrogen, Carlsbad CA, USA) supplemented with 15% fetal bovine serum (Biochrom, Merck Millipore, Darmstadt, Germany) and antibiotics (Penicillin-Streptomycin-Glutamine, Gibco/Invitrogen). Experiments were performed in antibiotics-free media with 1.5% l-glutamine (Gibco/Invitrogen). LCLs and all other cell lines were tested negative for *Mycoplasma* contamination.

#### CRISPR/Cas9-mediated knockout of *ABRAXAS1* in mammary epithelial cells

Parental MCF10A cells (ATCC®CRL-10317^TM^) used for the gene-editing were purchased from Sigma-Aldrich (Saint Louis, MO, USA) and were maintained according to the original protocol by Debnath and co-workers [77]. For experiments, cultivation relied on DMEM HAM’s F12 (PAA Laboratories GmbH, Pasching, Austria) with 5% horse serum (Gibco), MEM non-essential amino acids, 2 mM L-glutamine, 20 ng/ml EGF, 10 μg/ml Insulin, 100 ng/ml cholera toxin and 500 nM hydrocortisone.

In order to generate *ABRAXAS1* KO cell lines the method of Ran and coworkers [78] was followed. Briefly, the protospacer pair (5′-CACCGCTGAGGCGGCGGTAGCATGG-3′ and 5′-AAACCCATGCTACCGCCGCCTCAGC-3′) targeting the translation initiation codon of *ABRAXAS1* was first introduced into the *Bbs*I site in the pCAG-eCas9-GFP-U6-gRNA vector that was a gift from Jizhong Zou (Addgene plasmid #79145; https://n2t.net/addgene:79145; RRID:Addgene_79145). The vector construct and empty vector were transfected into parental MCF10A cells using Nucleofector II device with a kit L and program X-001 (Lonza, Basel, Switzerland) to create *ABRAXAS1-*KO and control cell lines, respectively. After 48 h, GFP positive cells were sorted with BD FACSAria^TM^ III (BD Biosciences, San Jose, CA, USA) and single cell colonies were expanded and screened. Genomic DNA was purified with Epicentre’s QuickExtract solution (Illumina, Madison, WI, USA) from each cell colony, followed by the amplification and sequencing of the target area with the primers 5′-CAGCAGAAGCGAAGGAGGAG-3′ (forward) and 5′-CAGCAGAAGCGAAGGAGGAG-3′ (reverse). Several cell clones with a disrupted ATG codon were obtained and the clones *ABRAXAS1*-KO-D1 (D1) and *ABRAXAS1*-KO-E7 (E7) as well as control cell line *ABRAXAS1*-CTRL-A9 (Supplementary Fig. S8a) were selected for further analyses. Passage-matched isogenic cell lines were used in each experiment.

#### ABRAXAS1 expression plasmid and site-directed mutagenesis

Wild-type *ABRAXAS1* carrying an N-terminal HA/FLAG-tag was cloned from pOZ-N-*ABRAXAS1* vectors into pcDNA3.1/Hygro(-) as previously described [57]. To generate expression plasmids for *ABRAXAS1* c.1106dup and *ABRAXAS1* c.577C>T encoding ABRAXAS1 p.(Ser370Ilefs*2) and ABRAXAS1 p.(Arg193*), respectively, the QuikChange Lightning Site-Directed Mutagenesis Kit (Agilent Technologies, Waldbronn, Germany) was applied according to the manufacturer’s instructions. The ABRAXAS1 expression plasmid (pcDNA3.1-ABRAXAS1) was used as plasmid DNA template after isolation from a DAM methylase positive *Escherichia coli* strain. Primers containing the desired mutations were: 5′-GTTAGATACACAAGACAAACGGATCTAAAGCAGATACTGGTAG-3′ (forward) and 5′-CTACCAGTATCTGCTTTAGATCCGTTTGTCTTGTGTATCTAAC-3′ (reverse) for pcDNA3.1-ABRAXAS1 c.1106dup and 5′-TGTCCACTGGTTTTAGCTGAGCAGTACAAACACAC-3′ (forward) and 5´-GTGTGTTTGTACTGCTCAGCTAAAACCAGTGGACA-3′ (reverse) for pcDNA3.1-ABRAXAS1 c.577C>T, which were purchased from Eurofins MWG Synthesis GmbH (Ebersberg, Germany) or from Biomers (Ulm, Germany).

#### DSBR-pathway analysis

Determination of DSBR-frequencies in LCLs followed the protocol established in [31] including guidelines and quality controls. Briefly, plasmid DNA mixtures of 30 μl containing 10 μg of one of the DSBR substrates (HR-EGFP/5′EGFP for HR, EJ5SceGFP for NHEJ, EJ-EGFP for microhomology-mediated end-joining (MMEJ), 5′EGFP/HR-EGFP for SSA) [79, 80] together with 10 μg of the meganuclease expression plasmid pCMV-I-SceI and 10 μg of the filler plasmid pBS (pBlueScriptII KS; Stratagene, Heidelberg, Germany) were transfected into 2–4 × 10^6^ LCL cells using electroporation cuvettes and a Gene Pulser with Pulse Controller (Bio-Rad Laboratories, München, Germany) at 200 V and 1050 μF (Bio-Rad). Subsequently, the transfected cells were transferred into fresh medium without antibiotics and cultivated for 48 h. Reconstitution of wild-type enhanced green fluorescent protein (EGFP) was determined by FACS analysis of the fraction of green fluorescent cells identified by gating in the FL1/FL2 dot plots (laser excitation at 488 nm) within the life cell-population in the side scatter/forward scatter dot plots (SSC/FSC gate) (FACS Calibur FACScan, BD BioSciences, Heidelberg, Germany). To determine transfection efficiencies, wild-type EGFP expression plasmid was used instead of the filler plasmid pBS in split samples each. Therefore, each quantification of green fluorescent cells monitoring DSBR was normalized using the individually determined transfection efficiency to calculate the DSBR-frequency.

To measure DSBR-frequencies in the chromatin context, K562 leukemia cells with chromosomally integrated reporter substrate HR-EGFP/3′EGFP, i.e. K562(HR-EGFP/3′EGFP) cells [80], were subjected to electroporation with 10 μg pCMV-I-SceI and 40 μg expression plasmid for one of the *ABRAXAS1* variants each. DSBR-frequencies were measured 72 h after transfection by FACS.

#### Transfection of LCLs and MCF10A cells for expression of exogenous ABRAXAS1 variants

For additional DSBR measurements, immunofluorescence microscopy and PLA LCLs were (co-)electroporated with 40 μg of expression plasmid for wild-type *ABRAXAS1*, the variants c.1106dup or c.577C>T or empty vector pcDNA3.1/Hygro(-) and cultivated for 24 or 48 h. For DNA fiber and cell cycle assays, western blotting and RT-qPCR, we used the nucleofection protocol with Amaxa B cell Nucleofector Solution (Lonza, Basel, Switzerland) for introduction of 10 μg expression plasmid for one of the *ABRAXAS1* variants.

For immunofluorescence microscopy, cell cycle and western blot analysis, MCF10A parental cells and clonal derivatives were nucleofected with 10 μg of expression plasmid for each ABRAXAS1 or control plasmid using Cell Line Nucleofector kit V using nucleofector program T-024 (Amaxa/Lonza) and cultivated for 24 h.

#### Immunofluorescence microscopy and proximity ligation assay (PLA)

For in situ analysis of DSBR cells were exposed to 2 Gy of ionizing radiation (Cs-137, GSR D1, Gamma-Service Medical GmbH, Leipzig, Germany), re-cultivated in fresh medium for 2 h (MCF10A clones) or 4 h with harvest by cytospinning on slides covered by poly-L-Lysine (Sigma-Aldrich, Steinheim, Germany) (LCLs). Depending on the specific antibody applied cells were pre-extracted or directly fixed with 3.7% formaldehyde and permeabilized with 0.5% TritionX-100. Five percent Goat Serum (Invitrogen) in PBS was utilized for blocking. Primary antibodies used were rabbit polyclonal antibodies targeting 53BP1 (NB100-304, Novus Biologicals, Littleton, CO, USA), MRE11 (NB100-142, Novus Biologicals) and RAD51 (H-92, Santa Cruz Biotechnology, Heidelberg, Germany) as well as mAbs against ABRAXAS1 (rabbit mAb 139191, clone EPR6310, Abcam, Cambridge, UK, recognizing the C-terminal fragment aa 394–408), BRCA1 (mouse mAb, clone MS110, Calbiochem, Darmstadt, Germany), γH2AX (mouse mAb, Ser139, clone JBW301, Millipore, Billerica, MA, USA) and p34 subunit of RPA (mouse mAb, clone 34–19, Calbiochem, Darmstadt, Germany). Secondary antibodies were AlexaFluor488- and AlexaFluor555-labeled from Invitrogen, Karlsruhe, Germany. Immunostained cells were embedded in VectaShield mounting media containing DAPI (4′,6-Diamidino-2-phenylindole dihydrochloride, Vector laboratories, Burlingame, CA, USA). In controls for simultaneous stainings with two primary antibodies cells were stained with each primary antibody together with the secondary antibody of the other primary antibody. For PLA Duolink^®^ PLA Reagents (Sigma) were used according to the manufacturer’s instructions and engaging primary anti-ABRAXAS1, anti-MRE11 and anti-BRCA1 antibodies. For each experiment negative controls were analyzed, i.e. without the primary antibodies or without the PLA probes. Nuclear immunofluorescence signals were imaged with the Keyence BZ-9000 microscope (Keyence, Neu-Isenburg, Germany) using a 100× objective and applying the same exposure times as well as intensity and minimal focus size thresholds throughout one experimental set. Automated identification and quantification of foci were carried out using BZ-II Analyzer software.

#### DNA fiber spreading assay

Fourty-eight hours after transfection of the control LCL BR-0968 with expression plasmids for different *ABRAXAS1* variants, we labeled cells with 20 µM 5-chloro-2-deoxyuridine (CldU, Sigma-Aldrich) for 30 min, followed by centrifugation and incubation in medium without (mock) or with 0.5 mM hydroxyurea (HU, Sigma-Aldrich) for 30 min essentially as described [35]. Hydroxyurea containing medium was again removed by centrifugation followed by incubation with 200 µM 5-Iodo-2 deoxyuridine (IdU, Sigma-Aldrich) for 30 min. Subsequently, cells were harvested, spotted, lysed, fixed and stained for immunofluorescence microscopy using the following primary antibodies: anti-BrdU (mouse, mAb, clone B44, 347580, BD BioScience) for detection of IdU and anti-BrdU (rat, mAb, clone BU1/75 [ICR1] NB500-169, Novus) for detection of CldU after blocking with 5% BSA. As secondary antibodies we used AlexaFluor555 (anti-mouse) and AlexaFluor488 (anti-rat) from Invitrogen (Darmstadt, Germany). The length of DNA fiber tracks was measured with a Keyence BZ-9000 microscope and BZ-II Analyser software (Keyence Germany GmbH, Neu-Isenburg, Germany).

#### Cellular survival and cell cycle distribution

For assessment of cell viabilities, LCLs were treated with different concentrations from 1 μM-2 mM of the PARP-inhibitor 1,5-isoquinolinediol (IQD) for 6 days and the colorimetric MTT assay was performed as described [31]. Media were replaced with fresh media including the corresponding IQD concentrations every second day. Each data set was corrected for mock-treatment. Cell viability curves were plotted and IC50-values of individual cell lines and cohorts were calculated using GraphPadPrism software.

To analyze the distribution of cells in cell-cycle phases and quantification of dying cells with subG1-content, cells were collected by centrifugation (LCLs) or trypsinized (MCF10A derivatives 24 h post transfection), washed with PBS, resuspended with 0.5 ml PBS and gently fixed on ice (1:1-mixture of acetone and 80% ethanol; −20 °C). Fixed cells were washed twice with ice-cold PBS, resuspended in propidium iodide staining solution (freshly added 50 µg/ml RNAse A; 50 µg/ml propidium iodide from Sigma-Aldrich in PBS) and incubated for 30 min in the dark. After diluting the suspension with 100 ml PBS with 0.2% EDTA, the stained cells were analyzed in a FACS Calibur flow cytometer (BD Biosciences).

#### Western blotting experiments

For the detection of endogenous proteins in LCLs, cells were harvested by centrifugation, whole cellular lysates prepared, 20–80 μg of proteins electrophoresed by SDS-PAGE and transferred to PVDF membrane as described [31]. For detection of exogenous proteins in MCF10A parental cells and clonal derivatives immunoblotting was performed 24 h post transfection with ABRAXAS1 expression plasmids and 24 µg of proteins subjected to immunoblotting. Proteins were detected by the following primary antibodies recognizing ABRAXAS1 (rabbit mAb 139191, clone EPR6310, Abcam, recognizing the C-terminal fragment aa 394–408), BRCA1 (mouse mAb, clone MS110, Calbiochem), phospho-Ser33 RPA32 (rabbit pAb, A300-244A, Bethyl, Montgomery, TX, USA), RPA32 antibody (rabbit pAb, A300-246A, Bethyl), Anti-phospho KAP1 Ser824 (rabbit pAb, A300-767A, Bethyl), Anti-KAP1 (rabbit pAb, A300-275A, Bethyl), cleaved PARP1 (rabbit pAb, AB3620, Chemicon/Millipore, Darmstadt, Germany), GAPDH (mouse mAb, clone T9484, Abcam), α-Tubulin (mouse mAb, clone DM1A, Abcam), and HA-tag (mouse mAb 2367, clone 6E2, Cell Signaling Technology, Danvers, MA, USA). Secondary antibodies were horseradish peroxidase-labelled goat anti-mouse and goat anti-rabbit (Rockland, San Diego, CA, USA). Chemiluminescent signals of band intensities were visualized by Clarity^TM^ Western ECL Substrate (Bio-Rad Laboratories), quantified in the linear range following background signal subtraction and normalized to the corresponding loading controls (ChemiDoc^TM^ MP Imaging System and ImageLab Software 5.2.1, Bio-Rad).

#### Reverse transcription quantitative polymerase chain reaction (RT-qPCR)

To verify expression of exogenous *ABRAXAS1* variants in LCLs we performed RT-qPCR analysis 48 h post transfection with expression plasmids. Total mRNA was isolated using the RNeasy Mini Kit (Qiagen, Hilden, Germany) and reverse-transcribed with QuantiTect Reverse Transcription Kit (Qiagen). Subsequent qPCR was performed using a SensiFast Lo-Rox kit (Bioline, Luckenwalde; Germany) on a Viia7 RUO Thermocycler (Applied Biosystems, Foster City, CA, USA). *HA-ABRAXAS1*-specific mRNAs were detected using ABRAXAS HEX primers and probes from Biomers (Ulm, Germany; Sense primer: 5′-CAAGCTCGATGGAGGATA-3′, antisense primer: 5′-TTCCCCAAGAAGAAAACC-3′ and probe: 5′-CCTACGACGTGCCCGACTAC-3′). RPS17 FAM primer assay from Bio-Rad and TBP VIC primer assay from Perkin Elmer (Thermo Fisher Scientific Inc., Waltham, Massachusetts, MA, USA) were used as internal controls. The differences in target mRNA expression were calculated with the 2− ΔΔCt method, whereby corrections were made for the mean of both housekeeping genes *RPS17* and *TBP*.

To detect CRISPR/Cas9-mediated *ABRAXAS1* expression changes in MCF10A clones we performed RT-qPCR as above engaging qPCR primers from Biomers (Ulm, Germany) specific for exon 1 in *ABRAXAS1*, namely sense primer Abraxas Ex1-forward: 5′-CATGGAGGGGGAGAGTACGTC-3 (covering the CRISPR/Cas9 targeted start codon, underlined), antisense primer Abraxas Ex1-reverse: 5′-GTGTCCGAGTCCGTGTTGAG-3′ and probe Abraxas Ex1 Fam: 5′-CTCGGGCTTTGTGCTCGGCGCACT-3′. HSP90 and TBP VIC primer assays from Perkin Elmer (Thermo Fisher Scientific Inc., Waltham, Massachusetts, MA, USA) were used as internal controls.

### Statistical analyses

Statistically significant differences between mean values of DSBR-frequencies, nuclear foci scores, track lengths in nascent DNA synthesis or cell cycle-phase percentages were calculated using the software GraphPad Prism versions 8–9 (GraphPad, San Diego, CA, USA). Once statistical significance was established for a data set by Kruskal–Wallis test, non-parametric Mann–Whitney test for unpaired samples was applied. GraphPad Prism versions 8–9 was also used to generate cell viability curves, calculate IC50 values and statistical significances of the differences between the IC50-values by Extra sum-of-squares *F* test of Log IC50. *P < 0.05; **P < 0.01; ***P < 0.001; ****P < 0.0001.

## Supplementary information

Suppl figures and tables revised

Extended material revised

aj-checklist

## Data Availability

The datasets generated during and/or analysed during the current study are available from the corresponding author on reasonable request.
