# Supplementary material for: ABRAXAS1 orchestrates BRCA1 activities to counter genome destabilizing repair pathways—lessons from breast cancer patients
Source: Cell Death Dis. 2023 May 17;14(5):328. doi: 10.1038/s41419-023-05845-6 (PMC10192442; doi:10.1038/s41419-023-05845-6)
Supplement: Supplementary file 1 — Suppl figures and tables revised [file 41419_2023_5845_MOESM1_ESM.pdf]

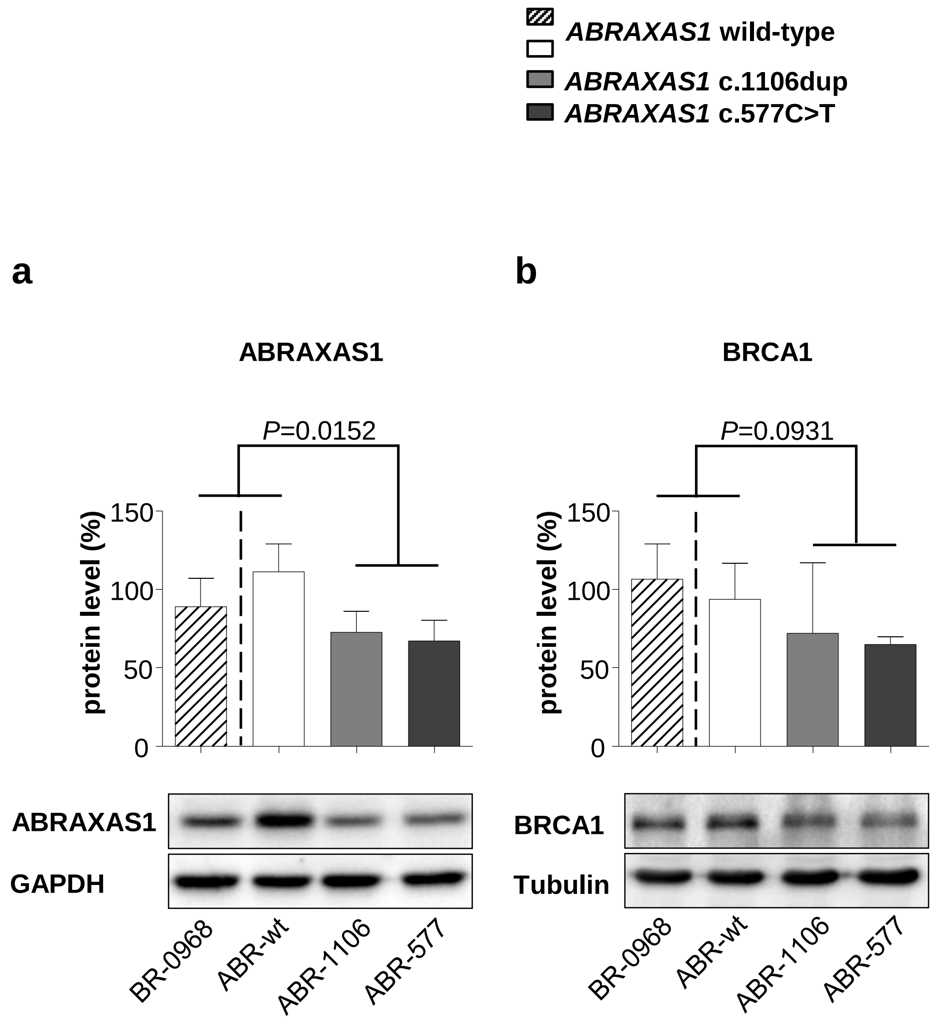

Supplementary Figure S1: Expression of DNA damage signaling proteins in *ABRAXAS1* mutation carriers and wild-type individuals.

Immunoblotting analyses were performed for two wild-type *ABRAXAS1* LCLs (external control BR-0968: hatched and ABR-wt: white columns), one LCL carrying *ABRAXAS1* c.1106dup (ABR-1106: light grey column) and one LCL carrying *ABRAXAS1* c.577C>T (ABR-577: dark grey column). The upper panels show quantification of protein levels from immunoblots (representative images in lower panels) using primary antibodies (**a**) anti-ABRAXAS1 (ab139191 from Abcam, recognizing the C-terminal fragment aa 394-408) and (**b**) anti-BRCA1 (MS110 from Calbiochem). Protein band intensities were quantified and normalized to the corresponding loading controls. Depending on the molecular weight of the detected protein GAPDH or α-Tubulin served as loading control. The mean value from wild-type LCLs (BR-0968 and ABR-wt) was set to 100% for each experimental day. Calculation of statistically significant differences between average mean values in LCLs with wild-type (BR-0968, ABR-wt) and mutated *ABRAXAS1* (ABR-1106, ABR-577) was performed via two-tailed Mann-Whitney U test. Columns show mean values obtained from three independent blotting experiments; bars, SD. Uncropped Western blots shown in Extended Figure E1.

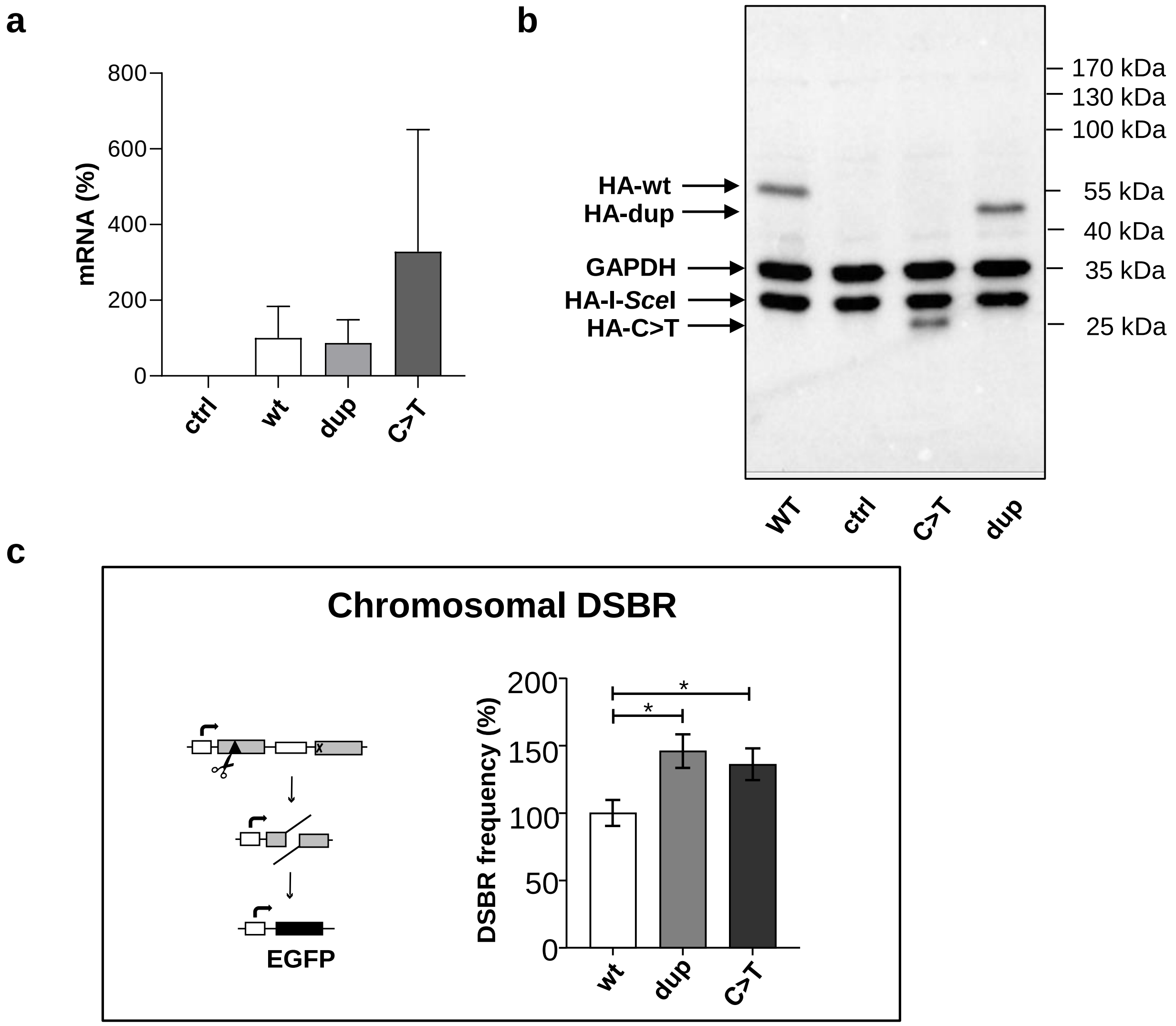

Supplementary Figure S2: Ectopic expression of *ABRAXAS1* variants and DSBR in the chromatin context.

(**a**) Ectopic expression of *ABRAXAS1* mRNA. LCL ABR-wt cells endogenously carrying wild-type *ABRAXAS1* were co-transfected with plasmid mixtures for DSBR measurements including I-*Sce*I expression plasmid (see Figure 3b) plus expression plasmid for wild-type ABRAXAS1 (wt), the mutated variants (dup, *ABRAXAS1* c.1106dup; C>T, *ABRAXAS1* c.577C>T) or empty vector (ctrl). Ectopic expression of *ABRAXAS1* variants in wild-type *ABRAXAS1* control LCLs was verified by RT-qPCR. mRNA expression levels 48h post-transfection are presented relative to cells expressing wild-type *ABRAXAS1* (100%). Columns show mean values from three independent experiments; bars, SEM;

(**b**) Ectopic expression of ABRAXAS1 protein. Western blot analysis using primary anti-HA antibody was performed for ectopically expressed HA-tagged ABRAXAS1 variant proteins and HA-tagged I-*Sce*I in K562(HR-EGFP/3´EGFP) cells 48h post-electroporation. Immunodetection of GAPDH served as loading control.

(**c**) DSBR in the chromatin context after ectopic expression of *ABRAXAS1* variants. Chromosomal DSBR (particularly SSA, left panel) was analyzed in K562(HR-EGFP/3´EGFP) cells 48h after cells had been co-electroporated with pCMV-I-SceI for cleavage of the chromosomally integrated DSBR reporter together with expression plasmids for wild-type *ABRAXAS1* (wt), *ABRAXAS1* c.1106dup (dup) and *ABRAXAS1* c.577C>T (C>T), respectively. Mean values from wild-type *ABRAXAS1* expressing cells were defined as 100% (corresponding to an absolute value of: 7.1×10^{-5}). Statistically significant differences were calculated between the mean values in cells ectopically expressing wild-type and mutated *ABRAXAS1* variants via Kruskal-Wallis-test followed by two-tailed Mann-Whitney U test. Mean values and SEM, n=12 from four independent experiments; * $P < 0.05$.

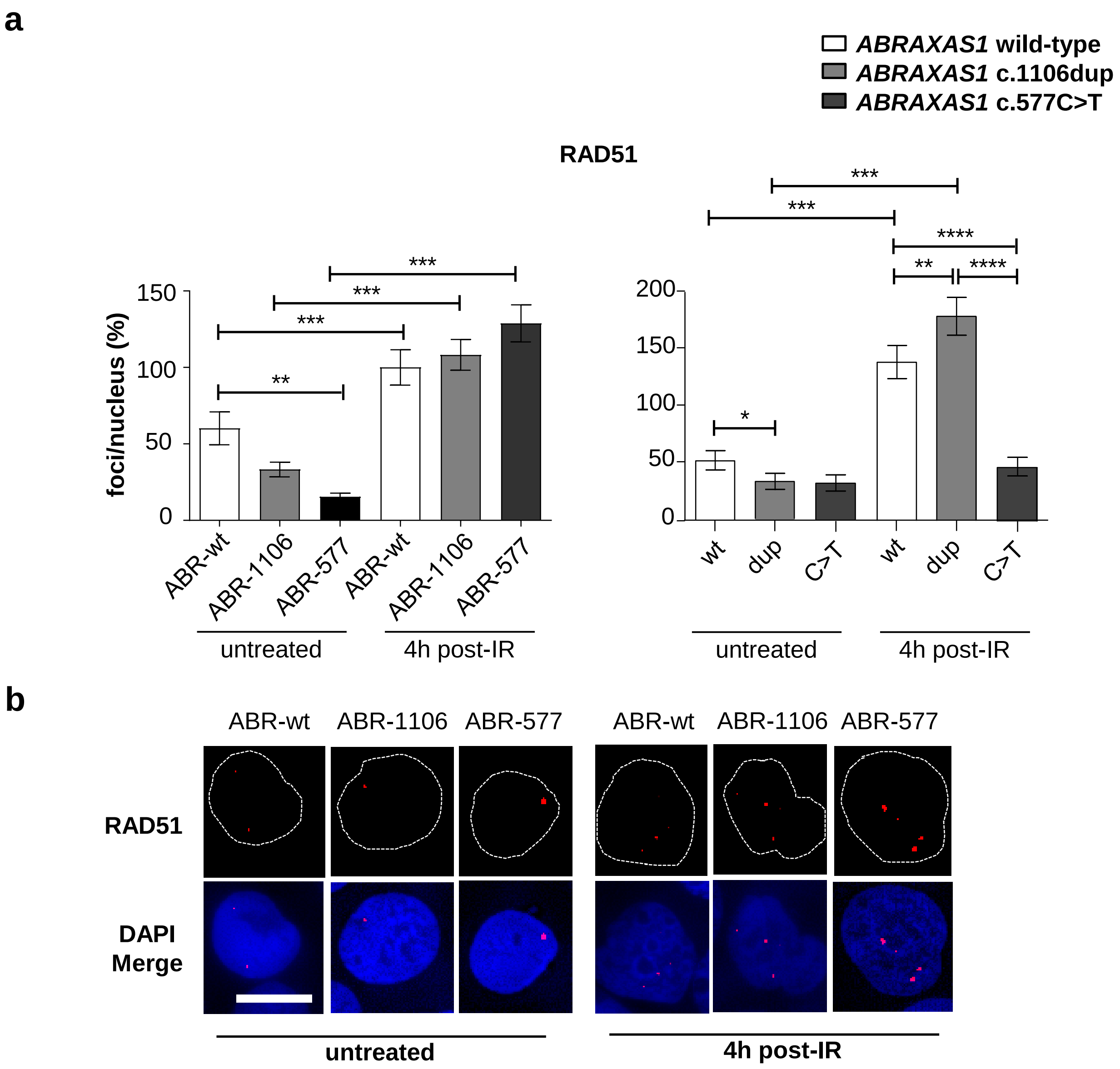

Supplementary Figure S3: Analysis of radiation-induced RAD51 foci accumulation in cells expressing wild-type versus mutated *ABRAXAS1*.

LCL cells were exposed to γ-irradiation with a dose of 2 Gy, immunostained 4h later and foci scored by automated quantification. Foci were normalized to the mean foci numbers of the wild-type *ABRAXAS1* cell line ABR-wt 4h post-IR measured on the same day. The reference value (100%) represents 3.3 RAD51 foci/nucleus. Data points indicate mean values and SEM of 150 nuclei obtained from three experiments. Statistically significant differences between mean foci values were calculated via Kruskal-Wallis-test followed by two-tailed Mann-Whitney U test. **P<0.01, ***P<0.001, ****P<0.0001.

(a) RAD51 foci quantification in LCL cells.

Left panel: Comparison of wild-type individual (ABR-wt: white columns), *ABRAXAS1* c.1106dup (ABR-1106: light grey column) and *ABRAXAS1* c.577C>T (ABR-577: dark grey column) mutation carriers.

Right panel: Comparison of ABR-wt cells ectopically expressing wild-type *ABRAXAS1* (wt), *ABRAXAS1* c.1106dup (dup) and *ABRAXAS1* c.577C>T (C>T) by transient transfection with expression plasmid followed by cultivation for 24h before irradiation.

(**b**) Representative images of RAD51 foci (red) in DAPI-stained nuclei (blue) from the wild-type ABR-wt, ABR-1106 and ABR-577. The scale bar represents 10 μm.

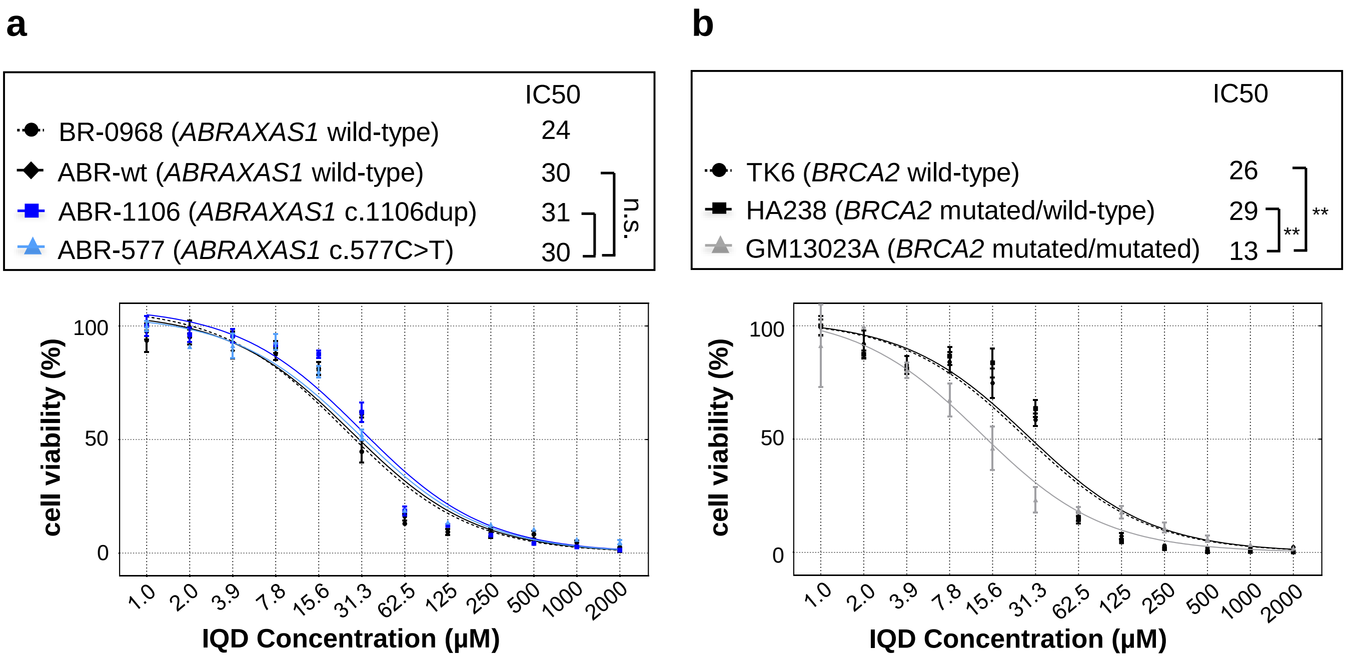

Supplementary Figure S4: Response to PARP-inhibition of cells from *ABRAXAS1* mutation carriers and wild-type individuals. To determine the cell viability after treatment with PARP inhibitor increasing 1,5-isoquinolinediol (IQD) concentrations (1 µM - 2 mM) were applied for 6 days. Media were replaced with fresh media including the corresponding IQD concentrations every second day. IC_{50}-values and SEM were calculated from survival curves for each cell line (n=4). Statistically significant differences between IC_{50}-values were determined using the Extra-sum-of squares F test. *P<0.05, **P<0.01.

(**a**) Comparison between LCLs with wild-type *ABRAXAS1* (ABR-wt: black rhombs), *ABRAXAS1* c.1106dup (ABR-1106: dark blue squares) and *ABRAXAS1* c.577C>T (ABR-577: light blue triangle); BR-0968 served as external wild-type *ABRAXAS1* control (black circles); n.s.: not significant.

(**b**) For comparison we analyzed three LCLs with wild-type *BRCA2* (TK6: black circles) as well as mono-allelically (HA238: black squares) and bi-allelically mutated *BRCA2* (GM13023A: grey triangles) demonstrating increased sensitivity to PARP inhibitor treatment upon complete loss of a functional *BRCA2* allele.

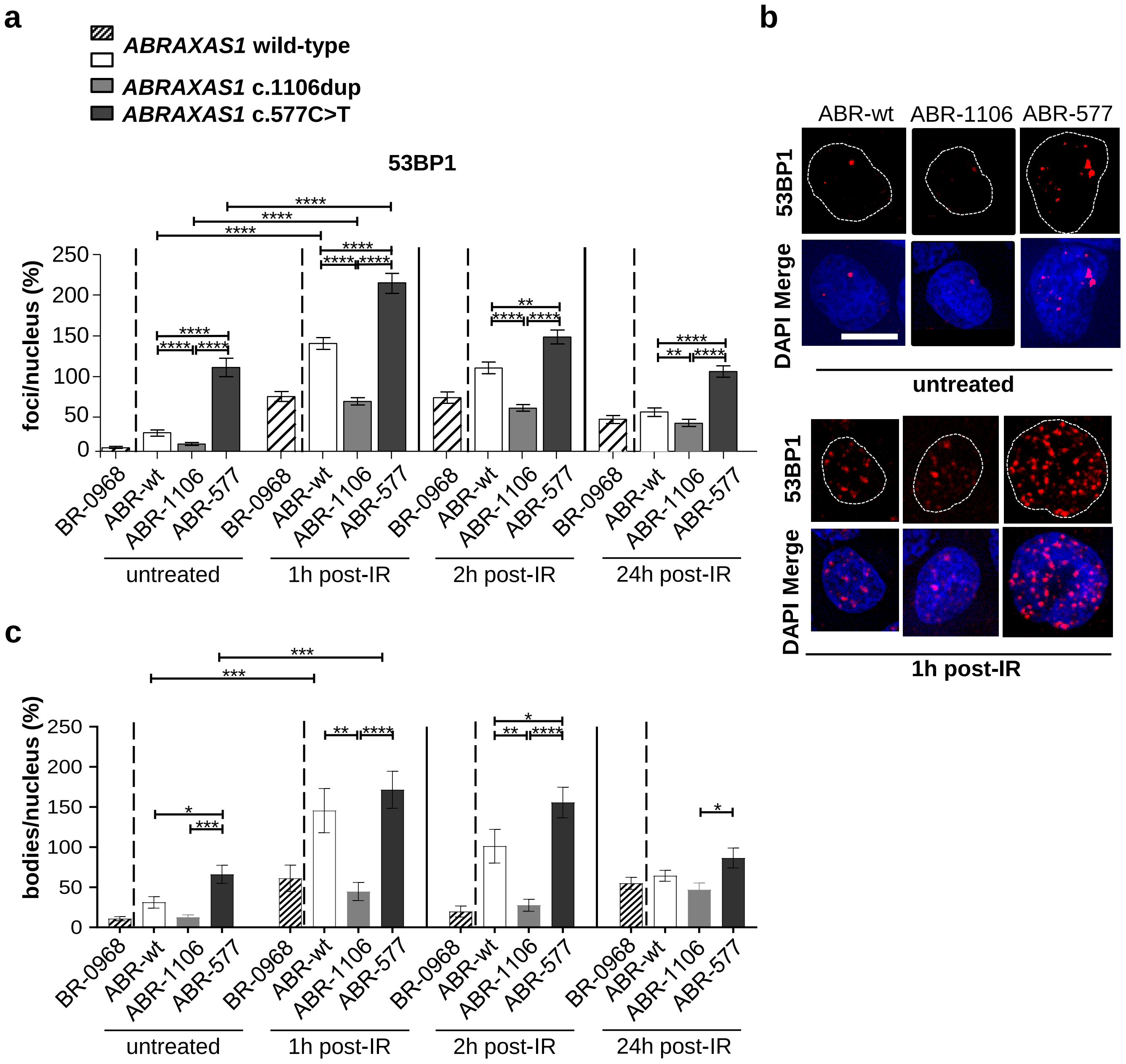

Supplementary Figure S5: Analysis of radiation-induced 53BP1 foci accumulation in *ABRAXAS1* mutation carriers and wild-type individuals.

(**a**) LCLs from wild-type *ABRAXAS1* individuals (external control BR-0968: hatched and ABR-wt: white columns), *ABRAXAS1* c.1106dup (ABR-1106: light grey column) and *ABRAXAS1* c.577C>T mutation carriers (ABR-577: dark grey column) were exposed to γ-irradiation (IR) with a dose of 2 Gy and re-cultivated before cells were fixed at the indicated time points. Foci values were scored by automated quantification of 50 nuclei for each time-point per experiment. Foci were normalized to the mean foci numbers of wild-type cell lines (ABR-wt and external control BR-0968) 1h post-IR measured on the same day. Reference value (100%) represents 10.7 53BP1 foci/nucleus. Statistically significant differences between foci values for LCLs ABR-wt, ABR-1106 and ABR-577 for each treatment condition and pairwise for untreated and irradiated cells (for reasons of clarity: 1h post-IR only) were calculated via Kruskal-Wallis-test followed by two-tailed Mann-Whitney U test. Data points indicate mean values and SEM of 150 nuclei obtained from three experiments. *P<0.05, **P<0.01, ***P<0.001, ****P<0.0001.

(**b**) Representative images of 53BP1 (red) in DAPI-stained nuclei (blue). The scale bar represents 10 μm.

(**c**) Re-evaluation of 53BP1 foci. Large 53BP1 bodies (3-8 μm^2) per nucleus were scored, normalized and statistically evaluated as in (a). Reference value (100%) represents one 53BP1 body/nucleus.

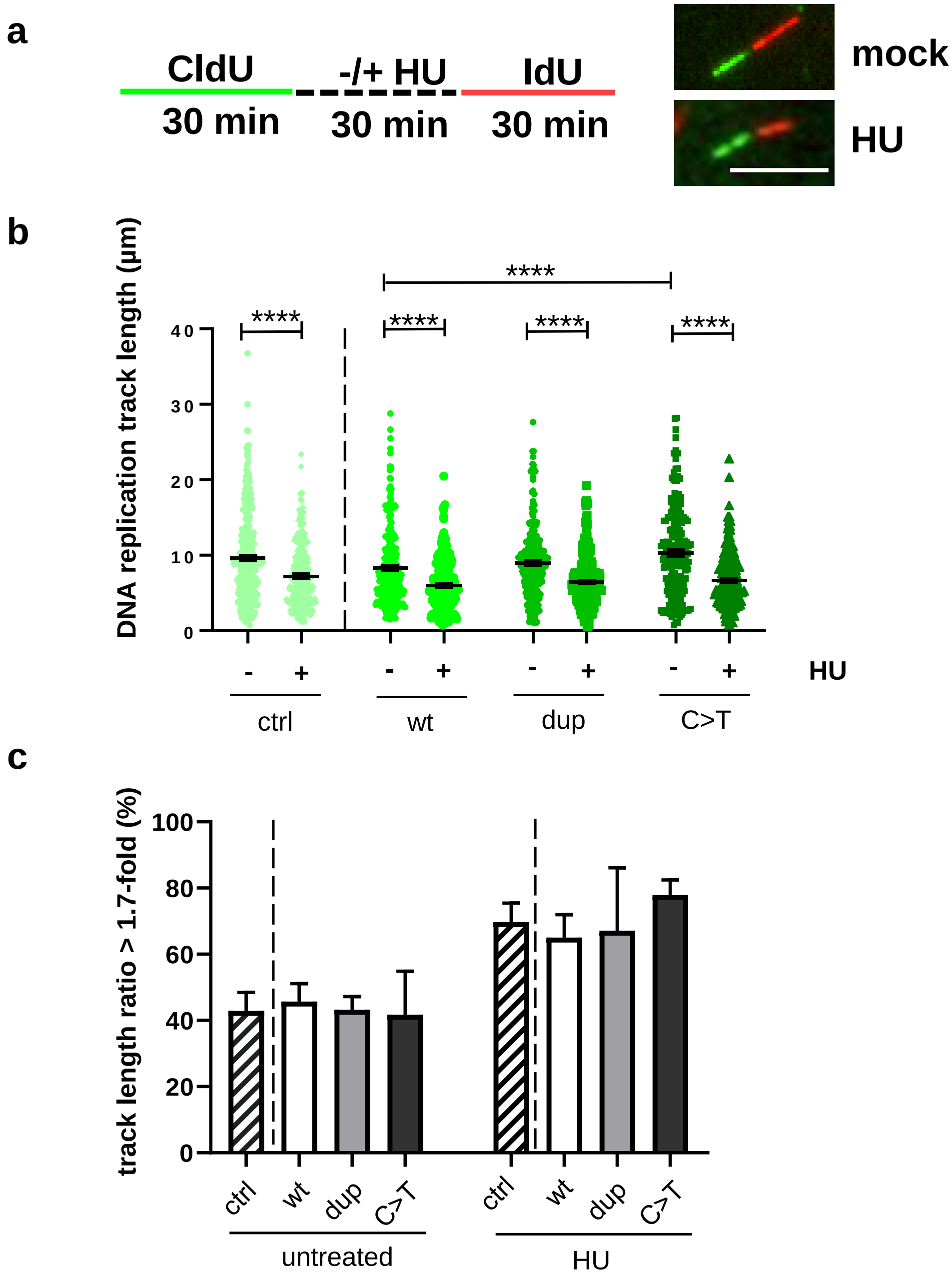

Supplementary Figure S6: Analysis of DNA replication after ectopic expression of *ABRAXAS1* variants.

Lymphoblastoid cells from the wild-type *ABRAXAS1* control individual were transfected with expression plasmids for wild-type *ABRAXAS1* (wt), the mutated variants (dup, *ABRAXAS1* c.1106dup; C>T, *ABRAXAS1* c.577C>T) or empty vector (ctrl). Fourty-eight hours after transfection cells were subjected to single DNA fiber analysis.

(**a**) Analysis scheme and representative fibers -/+ 0.5 mM HU are shown for ctrl. The size bar indicates 10 µm.

(**b**) CldU track length distributions of CldU- and IdU-double-positive DNA fibers following mock or HU exposure are graphically displayed. Mean values were calculated from fiber track lengths in two independent experiments with analysis of 98–158 single fibers in each sample. Statistically significant differences were calculated via Kruskal-Wallis-test followed by two-tailed Mann-Whitney U test. ****$p < 0.0001$;

(**c**) CldU/IdU track length ratios were calculated from the data in (b). Mean percentages of ratios exceeding 1.7-fold +/-SD are shown.

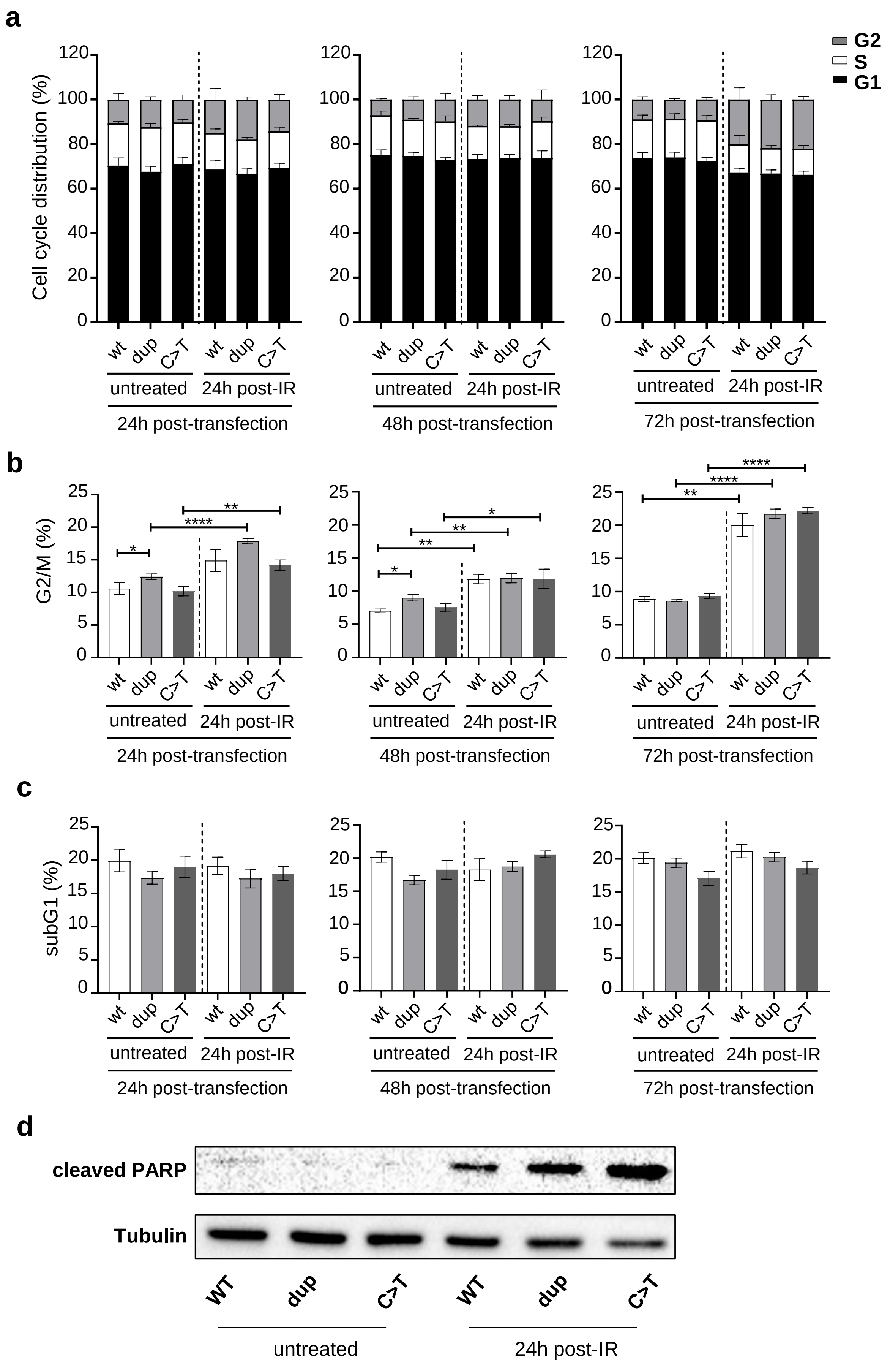
a
G2
S
G1
Cell cycle distribution (%)
wt
dup
C>T
untreated
24h post-IR
24h post-transfection
48h post-transfection
72h post-transfection
b
G2/M (%)
c
subG1 (%)
d
cleaved PARP
Tubulin
WT
dup
C>T
untreated
24h post-IR

Supplementary Figure S7: Cell cycle distribution and cell death of LCL cells after ectopic expression of *ABRAXAS1* variants.

Lymphoblastoid cells from the wild-type *ABRAXAS1* control individual were transfected with expression plasmids for wild-type *ABRAXAS1* (wt) and the mutated variants (dup, *ABRAXAS1* c.1106dup; C>T, *ABRAXAS1* c.577C>T) and cultivated for 24h, 48h or 72h before treatment with or without radiation (IR). DNA content was determined flow cytometrically after propidium iodide staining 24h post-IR. Mean percentages of cells were calculated from six to nine biological replicates obtained in two to three independent experiments, SEM. Statistically significant differences for mean values under each treatment condition and pairwise for untreated versus irradiated cells were calculated via Kruskal-Wallis-test followed by two-tailed Mann-Whitney U test for (b) and (c). *$P<0.05$, **$P<0.01$, ****$P<0.0001$.

(a) Distribution of living cells in the G1-, S- and G2/M-phases of the cell cycle.

(b) Percentages of living cells with G2/M DNA content.

(c) Percentages of cells with subG1 DNA content. Note that statistical analyses did not result in any significant differences.

(d) Analysis of PARP1 cleavage. Representative Western blot (n=2) showing immunodetection of cleaved PARP1 in control LCL transfected with expression plasmids for different *ABRAXAS1* variants followed by 48h of unperturbed growth, treatment (-/+ IR) and recultivation for 24h. Detection of Tubulin served as loading control. Uncropped Western blots are shown in Extended Figure E3.

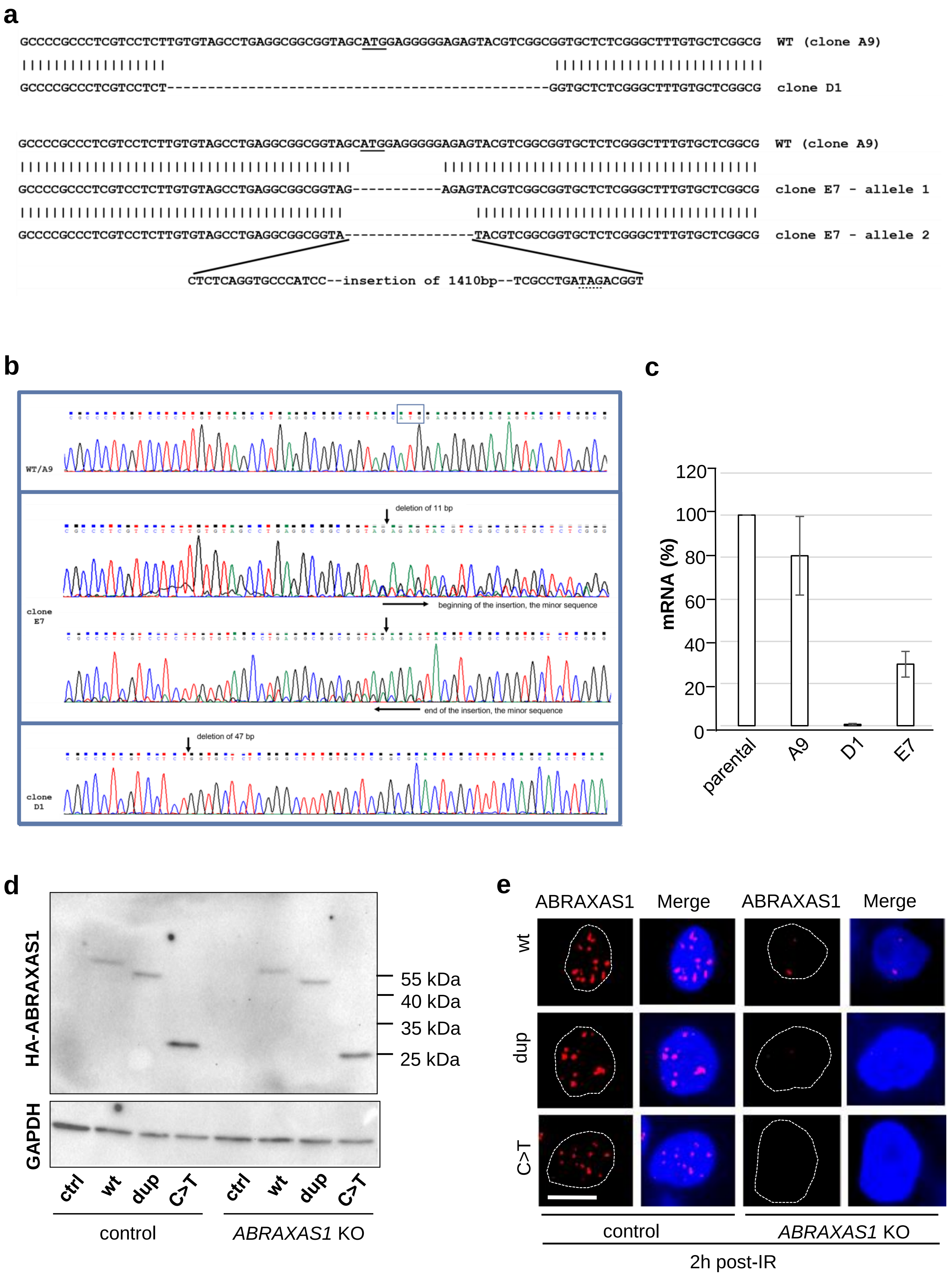
a
GCCCCGCCCTCGTCCTCTTGTGTAGCCTGAGGCGGCGGTAGCATGGAGGGGGAGAGTACGTCGGCGGTGCTCTCGGGCTTTGTGCTCGGCG WT (clone A9)
GCCCCGCCCTCGTCCTCT---GGTGCTCTCGGGCTTTGTGCTCGGCG clone D1
GCCCCGCCCTCGTCCTCTTGTGTAGCCTGAGGCGGCGGTAGCATGGAGGGGGAGAGTACGTCGGCGGTGCTCTCGGGCTTTGTGCTCGGCG WT (clone A9)
GCCCCGCCCTCGTCCTCTTGTGTAGCCTGAGGCGGCGGTAG-----------AGAGTACGTCGGCGGTGCTCTCGGGCTTTGTGCTCGGCG clone E7 - allele 1
GCCCCGCCCTCGTCCTCTTGTGTAGCCTGAGGCGGCGGTA----------------TACGTCGGCGGTGCTCTCGGGCTTTGTGCTCGGCG clone E7 - allele 2
CTCTCAGGTGCCCATCC--insertion of 1410bp--TCGCCTGATAGACGGT
b
WT/A9
deletion of 11 bp
clone E7
beginning of the insertion, the minor sequence
end of the insertion, the minor sequence
deletion of 47 bp
clone D1
c
mRNA (%)
120
100
80
60
40
20
0
parental
A9
D1
E7
d
HA-ABRAXAS1
GAPDH
55 kDa
40 kDa
35 kDa
25 kDa
ctrl
wt
dup
C>T
ctrl
wt
dup
C>T
control
ABRAXAS1 KO
e
ABRAXAS1
Merge
ABRAXAS1
Merge
wt
dup
C>T
control
ABRAXAS1 KO
2h post-IR

Supplementary Figure S8: Ectopic expression of *ABRAXAS1* variants in MCF10A cells with and without *ABRAXAS1 KO* and detection of nuclear signals.

(**a**) Sequence comparison between wild type (WT) control A9 and *ABRAXAS1*-mutated MCF10A clones E7 and D1. Upper panel: The out-of-frame deletion of 47 nucleotides destroys the translation initiation codon of *ABRAXAS1* (underlined) in one allele of clone D1. Sequence analysis indicated an even larger deletion in the other allele that prohibited identification by the PCR primers used. Lower panel: In one allele of clone E7 an out-of-frame deletion of 11 nucleotides encompassing the translation initiation codon was identified. In the other allele the translation initiation codon and surrounding sequences were found to be replaced by an insertion of 1410 nucleotides that is mostly derived from the all-in-one guide vector. A stop codon (dotted-underlined) close to the end of the insertion in the *ABRAXAS1* reading frame excludes the possibility of expression of a fusion protein containing part of ABRAXAS1.

(**b**) Chromatograms of the *ABRAXAS1* sequences in clones A9, E7 and D1. The chromatograms corresponding to the area encompassing the translation initiation codon in *ABRAXAS1* (boxed) visualize that neither clone E7 nor D1 contain an intact allele. The locations of the deletions are marked by arrows pointing downwards. The arrows pointing to the right and left show the beginning and end of the insertion in clone E7, respectively.

(**c**) Loss of expression of *ABRAXAS1* in *ABRAXAS1* KO cells was verified by RT-qPCR. mRNA expression levels in control MCF10A cells (clone A9) and *ABRAXAS1* KO cells (clones D1 and E7) are presented relative to parental MCF10A control cells (100%). Columns show mean values from two independent experiments; bars, SD;

(**d**) Western blot analysis was performed for HA-tagged ABRAXAS1 variant proteins using primary antibody anti-HA in parental MCF10A control and *ABRAXAS1* KO cells (clone D1). Immunodetection of GAPDH served as loading control. Cells were transfected with plasmid for expression of wild-type ABRAXAS1 (wt), the mutated variants (dup, *ABRAXAS1* c.1106dup; C>T, *ABRAXAS1* c.577C>T) or empty vector (ctrl).

(e) Representative images of ABRAXAS1 foci (red) in DAPI-stained nuclei (blue) corresponding to the quantification in Figure 6b, where MCF10A control and *ABRAXAS1* KO cells were transfected with plasmid for expression of wild-type ABRAXAS1 (wt) and the mutated variants (dup, *ABRAXAS1* c.1106dup; C>T, *ABRAXAS1* c.577C>T). The scale bar represents 10 μm.

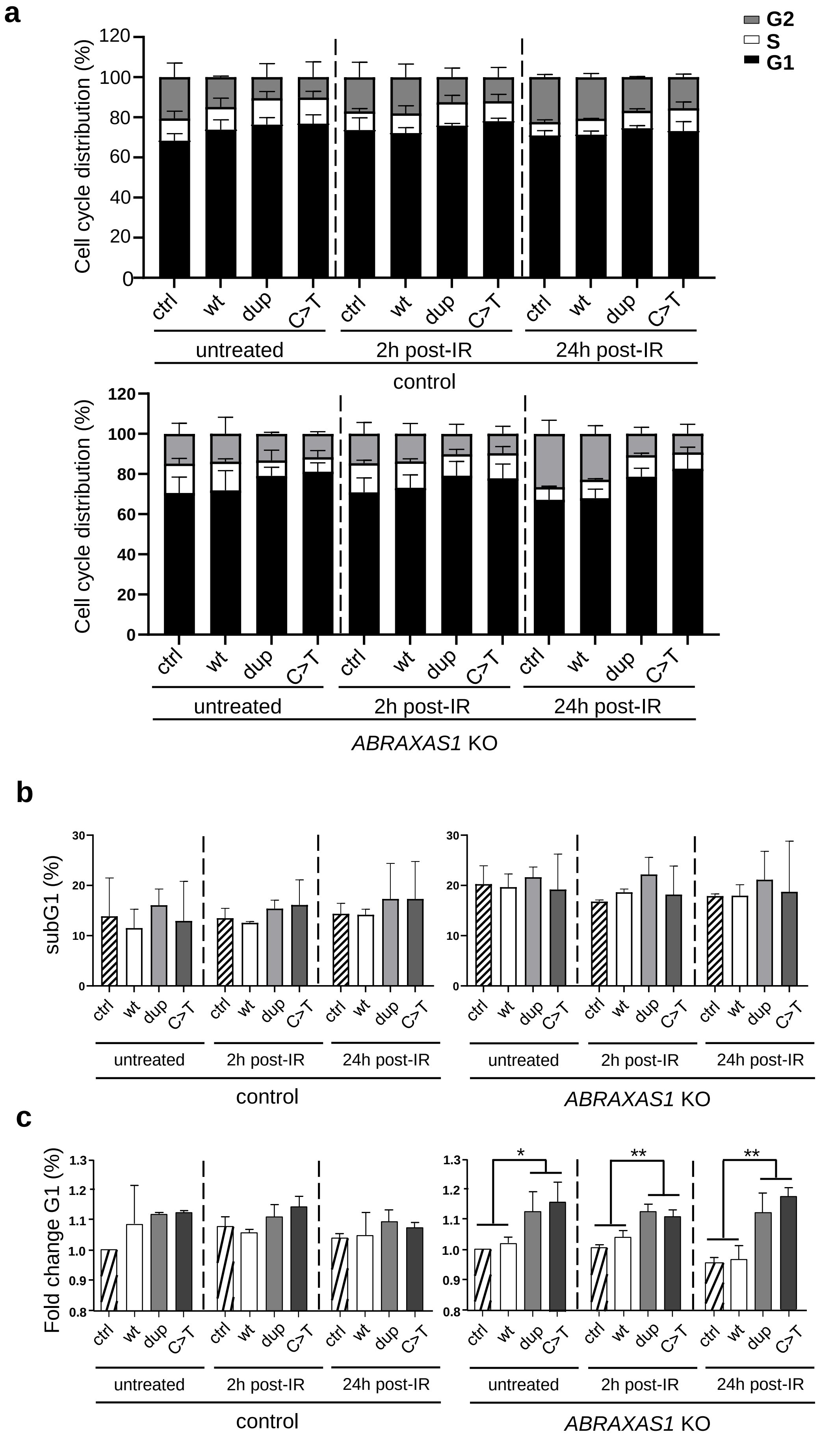

a
G2
S
G1
Cell cycle distribution (%)
120
100
80
60
40
20
0
ctrl
wt
dup
C>T
untreated
2h post-IR
24h post-IR
control
ABRAXAS1 KO
b
subG1 (%)
30
20
10
0
c
Fold change G1 (%)
1.3
1.2
1.1
1.0
0.9
0.8
*
**

Supplementary Figure S9: Cell cycle distribution and cell death of mammary epithelial cells after ectopic expression of *ABRAXAS1*.

DNA content was determined flow cytometrically after propidium iodide staining of control MCF10A (A9) and *ABRAXAS1* KO cells (D1) after expression of wild-type *ABRAXAS1* (wt), different *ABRAXAS1* variants (dup, *ABRAXAS1* c.1106dup; C>T, *ABRAXAS1* c.577C>T) or empty vector transfection (ctrl) with or without irradiation (IR) and recultivation for 2h or 24h post-IR. Mean percentages of cells were calculated from two independent experiments, SD.

(a) Distribution of living cells in the G1-, S- and G2/M-phases of the cell cycle.

(b) Percentages of cells with subG1 DNA content.

(c) Analysis of changes in the G1-phase distribution. Percentages of G1-phase cells were normalized to the percentage of untreated ctrl cells determined on the same day and fold changes calculated each. Statistical analysis was performed via t-test (paired, two-tailed) for differences between average mean fold changes in cells expressing no or wild-type (ctrl, wt) versus mutated *ABRAXAS1* (dup, C>T). *$P<0.05$, **$P<0.01$.

Supplementary Table S1. LCLs from German *ABRAXAS1* mutation carrier families.

Cell line	*ABRAXAS1* genotype	gene product	Health status	Age at diagnosis	Relationship
ABR-wt	wild-type	wild-type	healthy	n.a.	mother of ABR-1106
ABR-1106	c.1106dup	p.(Ser370Ilefs*2)	breast cancer	25	daughter of ABR-wt
ABR-577	c.577C>T	p.(Arg193*)	breast cancer	25	unrelated

n.a.: not analyzed.

Supplementary Table S2. Summary of results obtained with lymphoblastoid cells expressing German *ABRAXAS1* variants.

ABR genotype	Protein level	Nuclear foci post-IR							DSBR			DNA stress response					
	**ABR BRCA1**	**ABR**	**BRCA1 53BP1**	**γH2AX MRE11 RPA**	**RAD51**	**coloc. ABR / BRCA1**	**PLA ABR / BRCA1**	**PLA MRE11 / BRCA1**	**HR NHEJ**	**MMEJ**	**SSA**	**pKAP1 post-IR**	**survival (Ola)**	**fiber track length**	**pRPA**	**G2/M**	**MN post-IR**
**wild-type**	++	++	++	++	++	++	++	++	++	++	++	++	++	++	+	++	++
**c.1106dup**	+	+	+	++	++	+	+	+++	++	++	+++	++	++	++	++	++/+++[$]	+++
**c.577C>T**	+	++	+++	+++	++/+*	+	++	++	+++	++	+++	++	++	+++	+++	+++/++[§]	++

ABR, ABRAXAS1; coloc., co-localizing; DSBR, DNA double-strand break repair; fiber, DNA replication track length in the DNA fiber assay; IR, ionizing radiation treatment; MN, micronuclei; Ola, Olaparib sensitivity; pKAP1, phospho-KAP1; PLA, proximity ligation assay; pRPA, phospho-RPA;

* Note that in cells expressing *ABRAXAS1* c.577C>T as compared with wild-type *ABRAXAS1*, RAD51 foci decreased post-IR only when the variant was expressed transiently.

[$] Note that in cells expressing *ABRAXAS1* c.1106dup compared with wild-type *ABRAXAS1*, G2/M accumulation was observed only when the variant was expressed transiently.

[§] Note that in cells expressing *ABRAXAS1* c.577C>T compared with wild-type *ABRAXAS1*, G2/M accumulation was observed most pronounced when the variant was constitutively expressed in patient cells. Pronounced PARP cleavage post-IR was already seen when the variant was expressed transiently.
