# Supplementary material for: ABRAXAS1 orchestrates BRCA1 activities to counter genome destabilizing repair pathways—lessons from breast cancer patients
Source: Cell Death Dis. 2023 May 17;14(5):328. doi: 10.1038/s41419-023-05845-6 (PMC10192442; doi:10.1038/s41419-023-05845-6)
Supplement: Supplementary file 2 — Extended material revised [file 41419_2023_5845_MOESM2_ESM.pdf]

Extended Figure E1

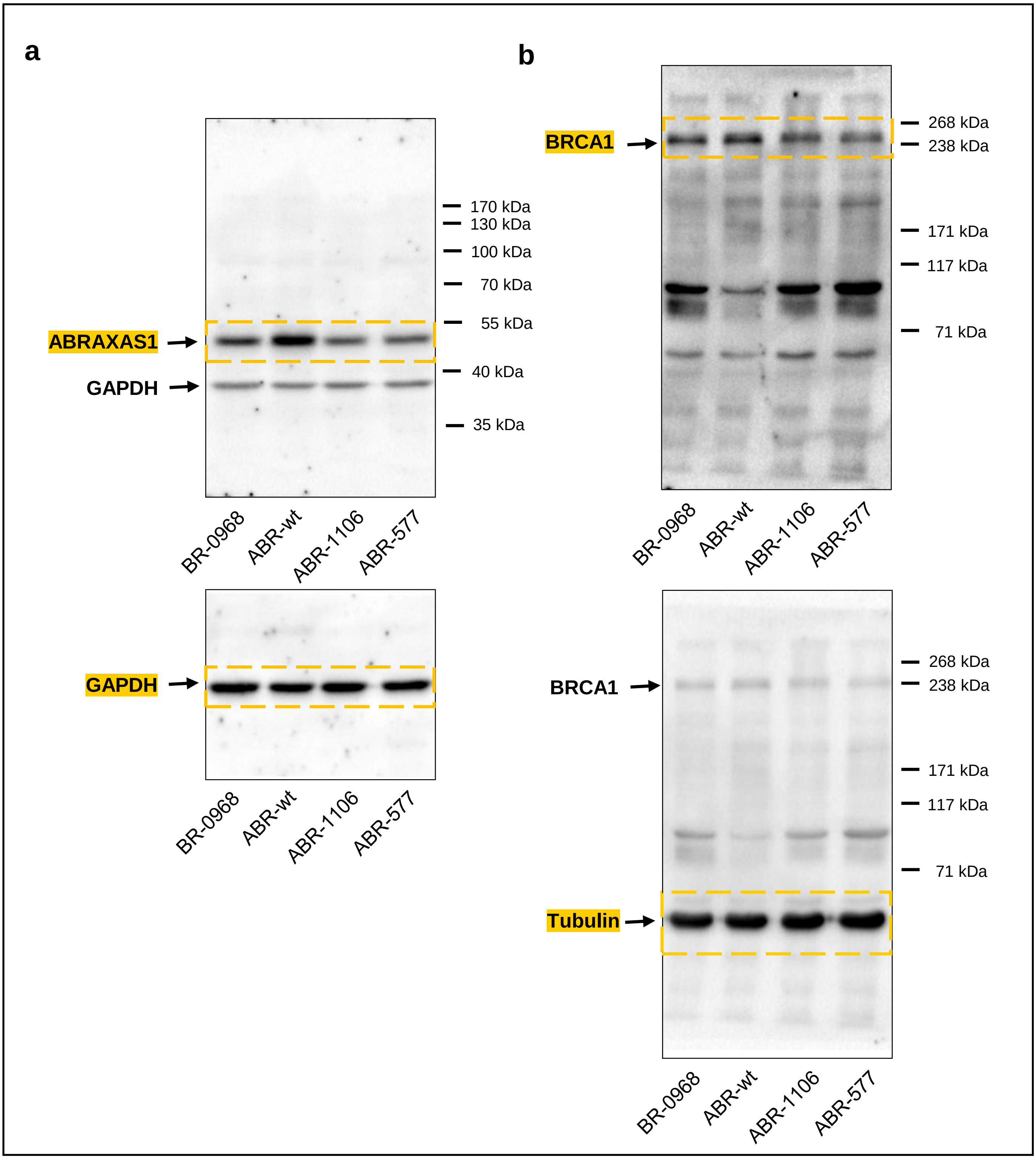

Fig E1: Original western blots of FigS1, bands shown in FigS1 are highlighted with yellow background. Yellow dashed lines on blots mark edges where blots were cut.
a: Original western blots of **Fig S1a**.
b: Original western blots of **Fig S1b**.

Extended Figure E2

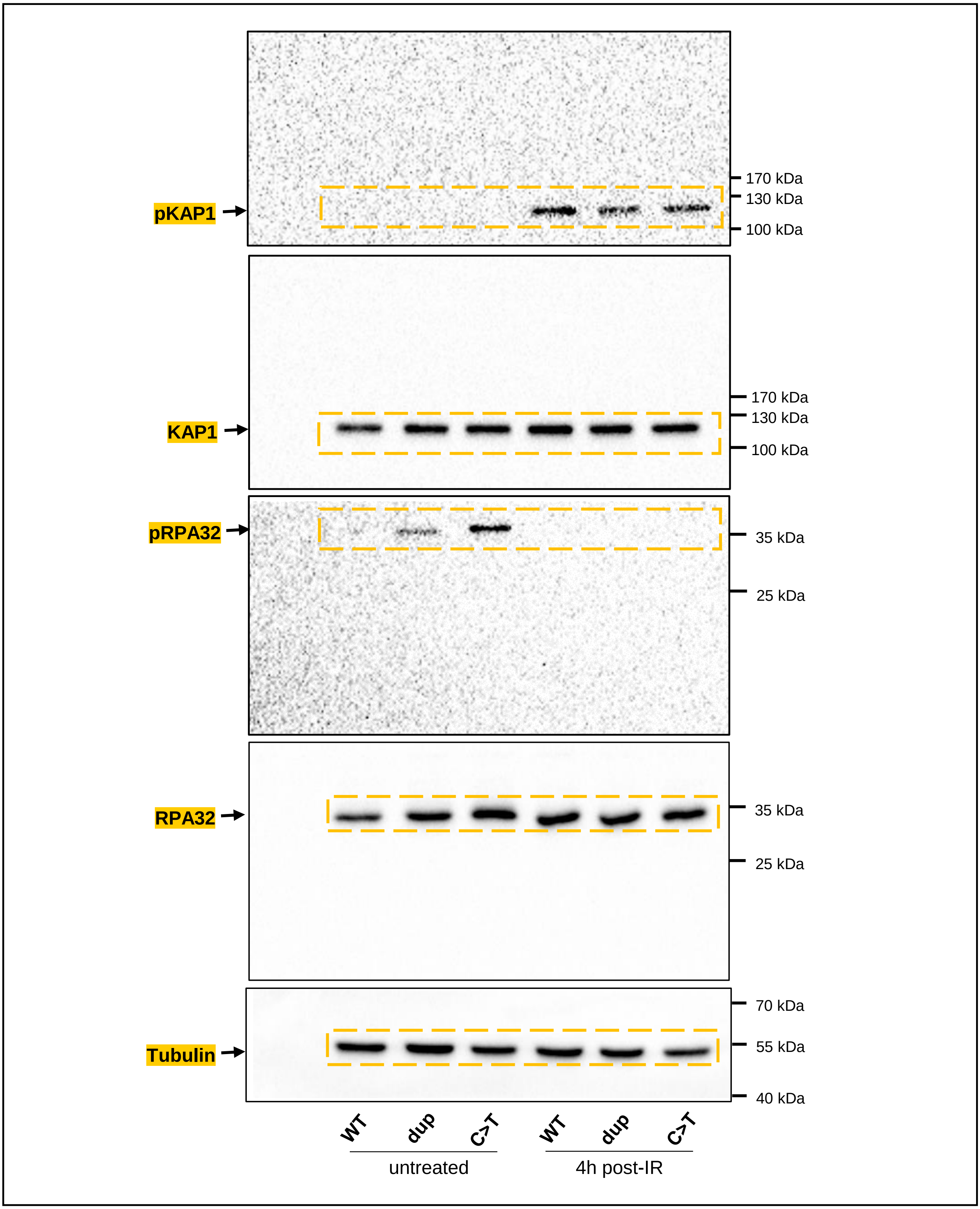

Fig E2: Original western blots of Fig4e, bands shown in Fig4e are highlighted with yellow background. Yellow dashed lines on blots mark edges where blots were cut.

Extended Figure E2

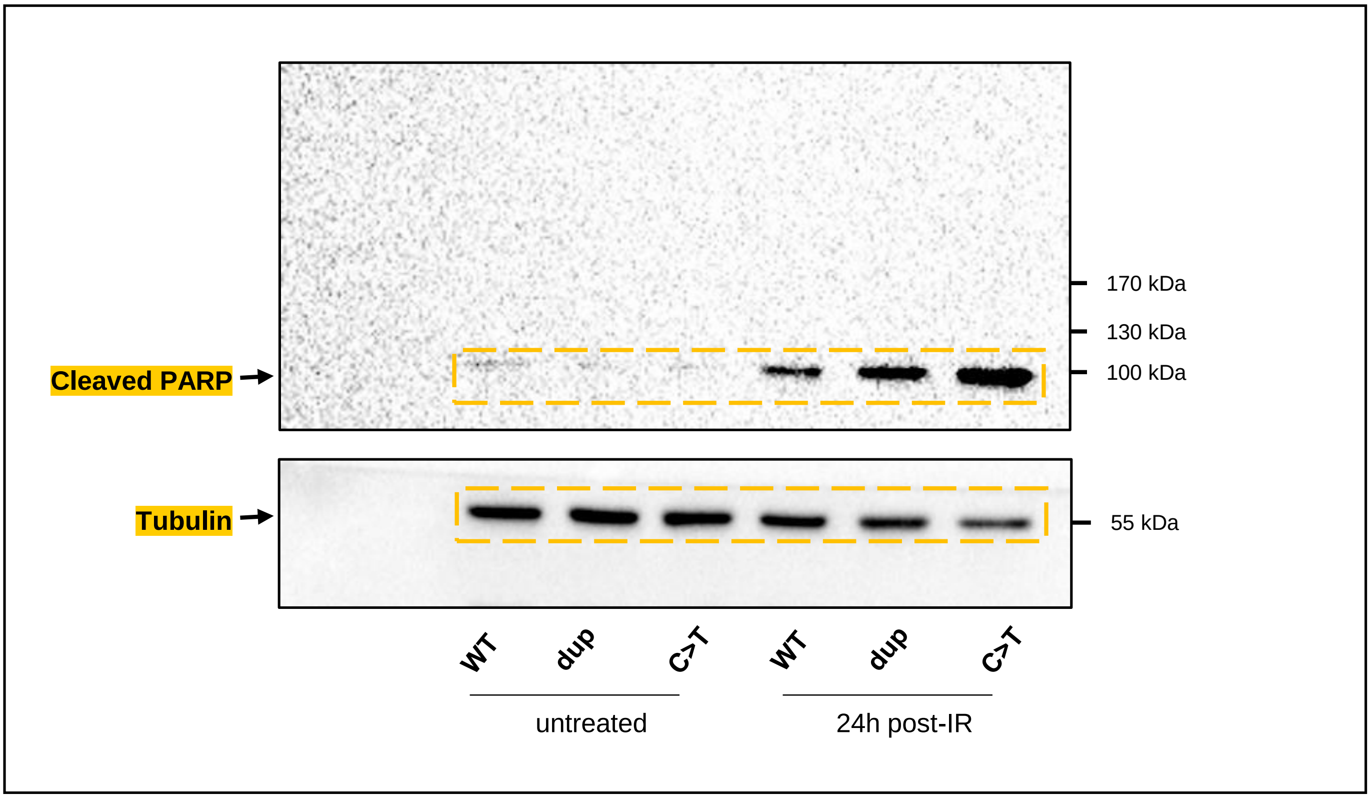

Fig E3: Original western blots of FigS7d, bands shown in FigS7d are highlighted with yellow background. Yellow dashed lines on blots mark edges where blots were cut.
